# A scoping review on efficacy and safety of medicinal plants used for the treatment of diarrhea in sub-Saharan Africa

**DOI:** 10.1186/s41182-023-00569-x

**Published:** 2024-01-03

**Authors:** Moitshepi T. A. Plaatjie, ThankGod E. Onyiche, Tsepo Ramatla, Johannes J. Bezuidenhout, Lesetja Legoabe, Nthatisi I. Nyembe, Oriel Thekisoe

**Affiliations:** 1 Unit for Environmental Sciences and Management, North-West University, Potchefstroom, South Africa; 2 Department of Veterinary Parasitology and Entomology, University of Maiduguri, Maiduguri, 600230 Nigeria; 3 Pharmaceutical Chemistry, School of Pharmacy, North-West University, Potchefstroom, 2520 South Africa; 4 Department of Zoology and Entomology, University of the Free State, Phuthaditjhaba, South Africa; 5 Gastrointestinal Research Unit, Department of Surgery, School of Clinical Medicine, University of the Free State, Bloemfontein, 9300 South Africa

**Keywords:** Medicinal plants, Sub-Saharan Africa, Diarrhea, Scoping review, Ethnobotanicals

## Abstract

**Background:**

In sub-Saharan Africa (SSA), significant morbidity and mortality have been linked to diarrhea, which is frequently caused by microorganisms. A rise in antimicrobial-resistant pathogens has reignited the search for alternative therapies. This scoping review aims to map the literature on medicinal plants in relation to their anti-diarrheal potential from SSA.

**Methods:**

Studies published from 1990 until April 2022 on medicinal plants used for the treatment of diarrhea from each country in SSA were searched on Scopus, Web of Science, Science Direct and PubMed. The selection of articles was based on the availability of data on the in vitro and/or in vivo*,* ethnobotanical*,* and cross-sectional studies on the efficacy of medicinal plants against diarrhea. A total of 67 articles (ethnobotanical (*n* = 40); in vitro (*n* = 11), in vivo (*n* = 7), cross-sectional (*n* = 3), in vitro and in vivo (*n* = 2) and ethnobotanical and in vitro (*n* = 2), were considered for the descriptive analysis, which addressed study characteristics, herbal intervention information, phytochemistry, outcome measures, and toxicity findings.

**Results:**

A total of 587 different plant species (from 123 families) used for diarrhea treatment were identified. Most studies were conducted on plants from the Fabaceae family. The plants with the strongest antimicrobial activity were *Indigofera* *daleoides* and *Punica* *granatum*. Chromatographic methods were used to isolate six pure compounds from ethyl acetate extract of *Hydnora* *johannis*, and spectroscopic methods were used to determine their structures. The majority of anti-diarrheal plants were from South Africa (23.9%), Ethiopia (16.4%), and Uganda (9%). This study highlights the value of traditional remedies in treating common human diseases such as diarrhea in SSA.

**Conclusion:**

Baseline knowledge gaps were identified in various parts of SSA. It is therefore recommended that future ethnobotanical studies document the knowledge held by other countries in SSA that have so far received less attention. Additionally, we recommend that future studies conduct phytochemical investigations, particularly on the widely used medicinal plants for the treatment of diarrheal illnesses, which can serve as a foundation for future research into the development of contemporary drugs.

**Supplementary Information:**

The online version contains supplementary material available at 10.1186/s41182-023-00569-x.

## Background

Diarrheal diseases rank as the third leading cause of infant and child mortality in developing countries, claiming the lives of approximately 1.8 million children annually [1] and remains high on the international public health agenda. Diarrhea is the passage of watery stools, usually at least three times in a 24 h period [2]. It is a common sign of gastrointestinal diseases brought on by a variety of pathogens, including bacteria, viruses, and protozoa [3]. Poor sanitation and hygiene are thought to be the root cause of 88% of deaths associated with diarrhea [4]. Researchers have linked the high incidence of diarrhea in sub-Saharan Africa (SSA) to poverty [5], an observation supported by its high morbidity and mortality in low-income communities in rural areas [6]. In 2015, the highest rates of child deaths from diarrheal illness were found in southern Asia and SSA. It is estimated that children under five experiences between 3.2 and 12 episodes of diarrhea each year in these regions [7].

Since the causative organisms are becoming more resistant, the antimicrobial medications that are currently accessible can be ineffective [8]. Moreover, some of the main diarrheal therapies (oral rehydration solutions) might not shorten the duration of the disease or reduce the volume of stool [9]. Therefore, a quest for novel and safe medications is ongoing. Plants are one possible source for the creation of novel drugs [10, 11]. Antimicrobial resistance, as well as limited access to conventional medicine, particularly in resource-poor countries, encourage most communities to rely on herbal medicine instead [12]. In any case, the World Health Organization (WHO) declared that traditional healing practices and medicinal plants for therapy continue to serve as the major source of healthcare for more than 80% of the emerging world population [13, 14]. Recently, James et al. reported that an estimated average of 58.2% of SSA populations rely on traditional, complementary and alternative medicine [15]. This is partly caused by the fact that most people cannot afford the expensive costs connected with the western health care system, as well as by people’s loyalty to their culture and traditions [16].

The Diarrheal Disease Control Programme of the WHO encourages the use of traditional folklore medicines in the control and management of diarrhea [17]. Several studies have suggested that oral transmission of knowledge from generation to generation, poor resource management, a lack of awareness of herbal medicine, and a lack of interest among the younger generation are all contributing to the loss of knowledge about medicinal plants [18–21]. Since traditional medical knowledge is passed down orally from generation to generation, it is possible for the fundamental details regarding some of the plants utilized, drug manufacturing techniques, diseases treatment, to be lost or forgotten during the process of knowledge transfer. As a result, documentation of traditional ethnomedicinal knowledge and herbal preparations for diarrhea can be used to preserve the knowledge and raise awareness about the need to conserve biological resources. Currently, no published scoping reviews focus on medicinal plants from SSA used for the treatment of diarrhea. Therefore, the purpose of this scoping review was three-fold: (i) to map the literature on medicinal plants in relation to their anti-diarrheal potential, (ii) to identify knowledge gaps in the primary literature regarding the efficacy of anti-diarrheal medicinal plants; (iii) and to ascertain specific areas of evidence where there is paucity on information to inform future study direction.

## Material and methods

### Study design

A scoping review was conducted according to the York Framework of scoping studies by Arskey and O’Malley [22] and Levac et al. [23], and further enhanced by the Preferred Reporting Items for Systematic Reviews and Meta-Analyses extension for scoping review (PRISMA-ScR) checklist [24], to collect data on medicinal plants used in treating diarrhea from each country of SSA. The proposed framework provides a systematic and standardised approach for developing scoping studies for new or broad questions of a complex or heterogeneous nature. Moreover, this method utilizes relevant databases and expands the range of study types to achieve a comprehensive view of the research available. A six-step process was used to conduct the scoping review namely, (a) setting the research questions, (b) sourcing studies, (c) selecting studies, (d) recording data, (e) summarizing, and (f) consulting on the findings. Due to the current policy that prohibits the registration of scoping reviews on PROSPERO, which is the International Prospective Register of Systematic Reviews, this review was not eligible for registration. PROSPERO serves as an online database specifically designed for the registration of systematic reviews and systematic review protocols.

### Research question

This review was conducted based on the primary research question "What is the current range of literature related to the potential application of medicinal plants against diarrhea, and to what extent has research explored their efficacy and safety?".

### Information sources and search strategy

Articles published in English from 1st January 1990 to 30 April 2022 were searched in four databases: Scopus, Web of Science, Science Direct, and PubMed. The search keywords included "diarrhea" and "humans" and (["plant" or "herb" or "traditional medicine"] or ["side effect" or "health effect" or "toxic" or "safety"]), along with the names of each country from SSA (Table 1). The closing time frame of the database search was the 19th of May 2022.

**Table 1:** Search strategy for published articles

S/No.	Source	Query/search string	Results
1.	Science Direct	(Diarrhea) and (humans) AND (“plant” or “herb” or “traditional medicine”) OR (side effect” or “health effect” or “toxic” or “safety”)	326
2.	PubMed	(Diarrhea) and (humans) AND (“plant” or “herb” or “traditional medicine”) OR (side effect” or “health effect” or “toxic” or “safety”)	270
3.	Scopus	(Diarrhea) and (humans) AND (“plant” or “herb” or “traditional medicine”) OR (side effect” or “health effect” or “toxic” or “safety”)	491
4.	Web of Science	(Diarrhea) and (humans) AND (“plant” or “herb” or “traditional medicine”) OR (side effect” or “health effect” or “toxic” or “safety”)	58

### Study selection

The relevant papers underwent an initial screening by evaluating their titles and abstracts. During this phase, specific keywords and phrases related to the study’s focus were scrutinized. The search involved terms such as "diarrhea," "humans," "plant," "herb," "traditional medicine," "side effect," "health effect," "toxic," and "safety." The inclusion criteria were applied to identify articles that directly addressed the research topic. Some articles lacked an abstract for preliminary review, necessitating a comprehensive assessment during the subsequent full review to determine their relevance. Potential full-text articles were downloaded to assess eligibility, and results were entered into a Microsoft Excel spreadsheet. Thereafter, a full-text evaluation of the downloaded articles was carried out. A meticulous full-text evaluation followed, wherein one author utilized predetermined eligibility criteria for screening. To enhance the robustness of the screening process, a second investigator independently verified the data, with any discrepancies resolved through consultation with a third reviewer.

This systematic approach, incorporating targeted keyword searches and rigorous screening processes, was employed to identify and select papers that align closely with the research objectives.

### Inclusion and exclusion criteria

Articles were included based on the following predefined eligibility criteria: (i) full-text articles published in the English language, (ii) in vitro and/or in vivo*,* ethnobotanical study*,* and cross-sectional studies on the efficacy of medicinal plants against diarrhea, (iii) detailed information about the plant (for instance, scientific name and plant parts used), (iv) geographical location of origin of the plant was clearly provided. Studies were excluded if they were (i) not conducted in SSA, (ii) lacking information on medicinal plants, (iii) not reporting information about anti-diarrheal medicinal plants, including their concentration (iv) review articles or letters, (v) and articles not published between 1st January 1990 and 30th April 2022.

### Data extraction

The relevant data about SSA medicinal plants was extracted using a pre-designed Microsoft Excel format. Extracted data included (i) first author (ii) study characteristics: country, year, type of study (in vitro or in vivo ethnobotanical, and cross-sectional), (iii) herbal intervention information (plant part used, formation, traditional/botanical, doses, type of solvent used, and duration of treatment (iv) comparator: drugs (i.e. positive controls), (v) study population (gender, age, animal model, type weight of animal, diagnosis, and assay), (vi) outcome measures, and main findings, and (vii) others: funding details, limitations, and remarks.

### Synthesis

This scoping review did not examine the quality or methodology of bias adopted by the included articles Tricco et al. [25]. Due to the fact that scoping reviews are not intended to produce a critically appraised and synthesised answer to a specific question, but merely aim at giving an overview or map of all the evidence. As a result, it is normally not necessary to assess the methodological limitations or bias of evidence included in a scoping review (unless the objective specifically dictates otherwise) [26]. Using the study by Poswal et al. [27] as an example, the synthesis of information included a rate of recurrence analysis of key research themes, as well as a grouping of included and excluded studies. This meticulous data extraction approach ensured a nuanced understanding of the evidence landscape while maintaining transparency and completeness, in line with the PRISMA-ScR checklist [24] (see Additional file 1: Table S1). Graphical representations in the form of bar charts were generated to assess the extent to which included studies adequately addressed individual items pertaining to the checklist elements related to abstract and introduction, methods, results, discussion, and funding status.

## Results and discussion

### Study inclusion

**Fig. 1:**
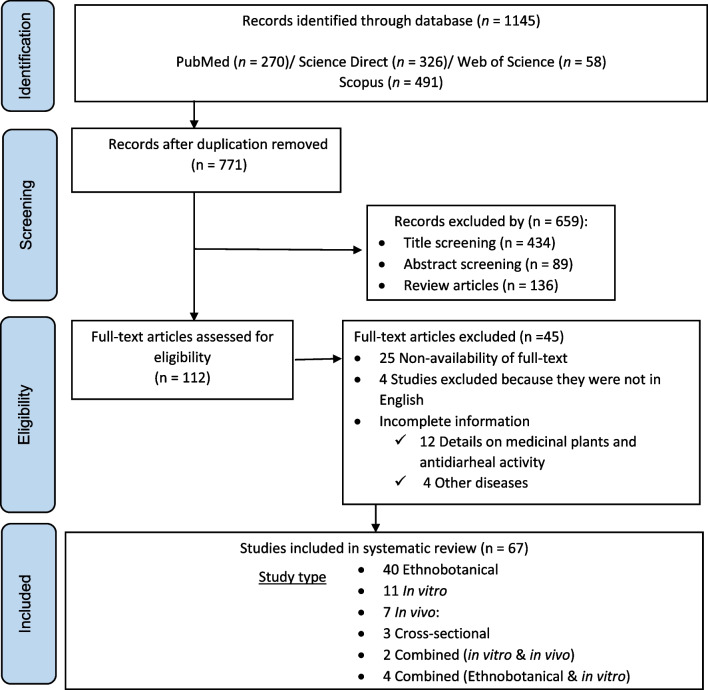
PRISMA flowchart of included studies

The PRISMA flowchart (Fig. 1) summarizes the search results and selection process in the present scoping review study. A total of 1145 [(PubMed, *n* = 270, Science Direct (*n* = 326), Web of Science (*n* = 58), and Scopus (*n* = 491)] articles were found from the initial database search. After removing duplicates (*n* = 374), 523 studies were excluded based on title and abstract. Reasons for exclusion for the other 45 articles include: (i) non-availability of full-text (*n* = 25); (ii) studies published in languages other than English (*n* = 4); (iii) incomplete information; on medicinal plants and anti-diarrheal activity (*n* = 12); and (iv) other diseases other than diarrhea (*n* = 4). One hundred and twelve full-text articles were assessed for eligibility, from which 45 were excluded. A total of 67 studies about medicinal plants from SSA used for the treatment of diarrhea were used in writing this review.

### Study characteristics

Of the 67 articles included in the review, exactly 40 reported ethnobotanical information, in vitro findings (*n* = 11), in vivo findings (*n* = 7), cross-sectional findings (*n* = 3), combined (both in vitro and in vivo studies; *n* = 2) and combined (ethnobotanical and in vitro findings; *n* = 4). All included studies were published between 2000 and 2022. The interest in using medicinal plants for the treatment of diarrhea research has increased over time, especially since 2008. Most studies were from South Africa (23.9%), Ethiopia (16.4%), and Uganda (9%). Other countries included Nigeria (7.5%) and Kenya (7.5%) (Table 2). There are numerous benefits from rapid publication rates, which include reducing the effects associated with diarrheal disease and medicinal plant research in SSA, but also throughout the world. South Africa, Ethiopia, and Uganda are still among the SSA countries with the highest percentage of gross domestic product allocated to research and development. Thus, the high rate of medicinal plant activity against diarrhea-related research outputs in these African countries explains the investment in research and development [28]. In the study, the analysis reveals the countries with the highest citations for medicinal plants used in the treatment of diarrhea. This could also be justified by the fact that these countries have a strong attachment to traditional medicine, which relies on plants as a primary ingredient, and there has been a recent increase in interest and reliance on indigenous medicinal plants in rural communities because of the high costs associated with conventional medicines [1]. Funding was public for 28 studies (41.8%), of which 13 received support from government sources, 5 received municipal funding, and 10 received private/industry funding. No funding was available for support for 2 studies (3%), funding was unreported for 37 studies (55.2%) (see Additional file 1: Tables S2, S3).

**Table 2:** Summary of studies included (n = 67)

Characteristics	Number of studies
Year of publication	
January 2000–December 2010	22 (32.8%)
October 2011–January 2022	45 (67.2%)
Study location	
South Africa	16 (23.9%)
Ethiopia	11 (16.4%)
Uganda	6 (9.0%)
Nigeria	5 (7.5%)
Kenya	5 (7.5%)
Mauritius	3 (4.5%)
Mozambique	3 (4.5%)
Tanzania	3 (4.5%)
Sudan	2 (2.9%)
Cameroon	2 (2.9%)
Congo	2 (2.9%)
Ghana	1 (1.5%)
Lesotho	1 (1.5%)
Mali	1 (1.5%)
Angola	1 (1.5%)
Zimbabwe	1 (1.5%)
Swaziland	1 (1.5%)
Malawi	1 (1.5%)
Benin	1 (1.5%)
Madagascar	1 (1.5%)

**Fig. 2:**
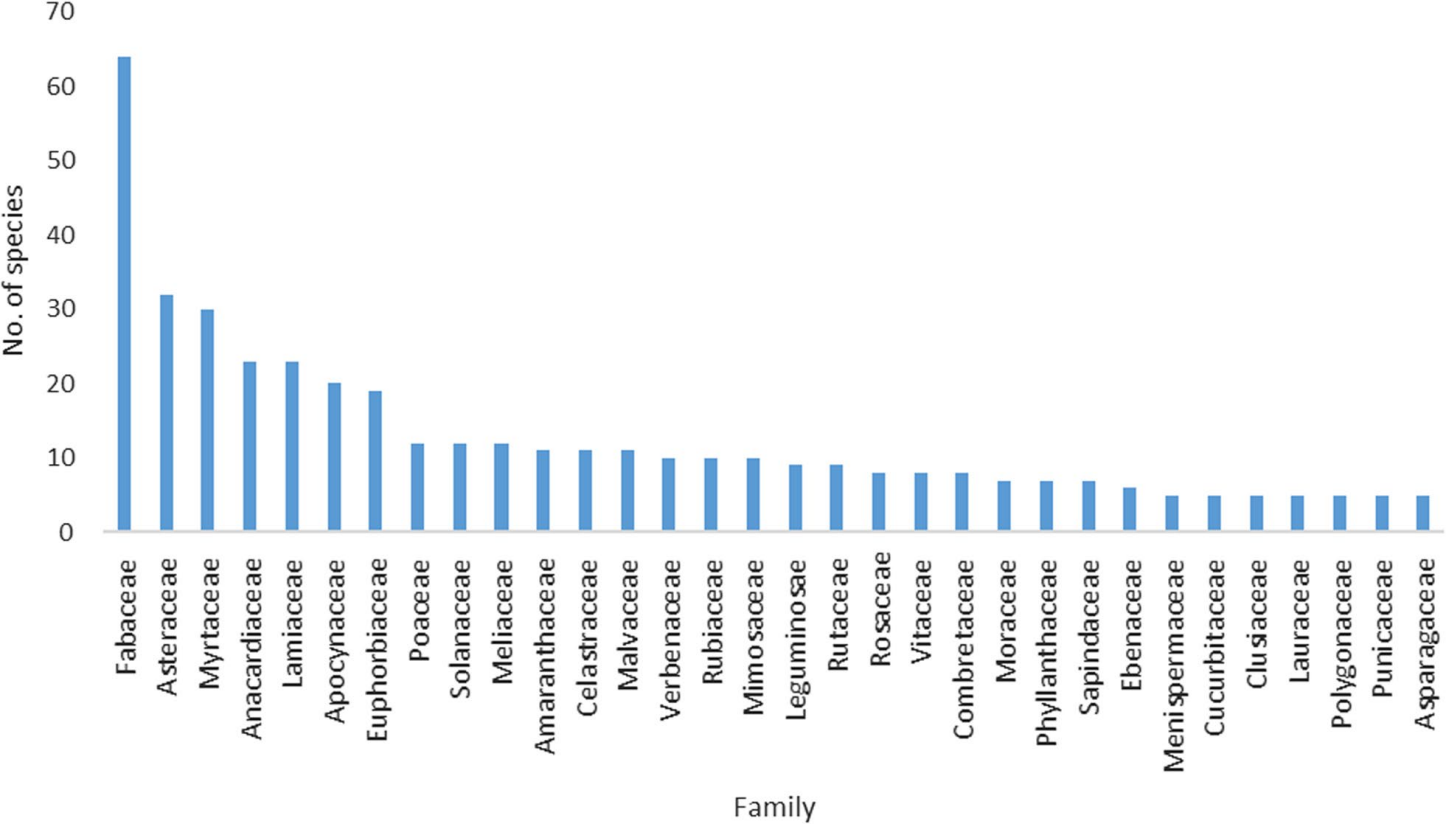
Family wide distribution of those families that contain at least five species

Eleven included in vitro studies mostly used the following test organisms: *Staphylococcus* *aureus,* *Escherichia* *coli,* *Salmonella* *typhimurium,* *Pseudomonas* *aeruginosa,* and *Enterococcus* *faecalis.* Among the 7 included in vivo studies, one study used both rabbit and mice model, three studies used rat models, and another three used mouse models. These findings are summarized in Additional file 1: Table S2. In the current study, the most frequent plant families among the medicinal plants include *Fabaceae* with 64 species, Asteraceae with 32 species, Myrtaceae with 30 species, Anacardiaceae and Lamiaceae (each with 23 species), Euphorbiaceae with 19 species, Poaceae, Solanaceae and Meliaceae (each with 12 species) (Fig. 2). Moreover, several studies have also documented Fabaceae and Asteraceae as dominant families in Nigeria, South Africa, Ethiopia, Congo, and Rwanda, in that order [12, 17, 29–31]. It is possible that the species richness of these families contributes to their dominance in medicinal plants [31].

Five hundred and eighty-seven (587) plant species were investigated in the current study. Of them, the most investigated were: *Psidium* *guajava* (*n* = 17), *Syzygium* *cordatum* (*n* = 8), *Sclerocarya* *birrea* (*n* = 6), *Gymnosporia* *senegalensis* (*n* = 5), *Ocimum* *gratissimum* (*n* = 5), and *Vernonia* *amygdalina* (*n* = 5). Increased interest in research on a particular plant species with antidiarrheal activity may denote higher bioactive phytochemicals in the plant and thus, a higher number of citations [32]. There is evidence that different plant species contribute to an ecosystem differently and that dominant species can shape community structure and diversity as they possess high biomass, high productivity, and other characteristics. It is important to have such evidence at hand when prioritizing future pharmacological research agendas [32].

### Specific objective addressed by studies

#### Interventions used

**Fig. 3:**
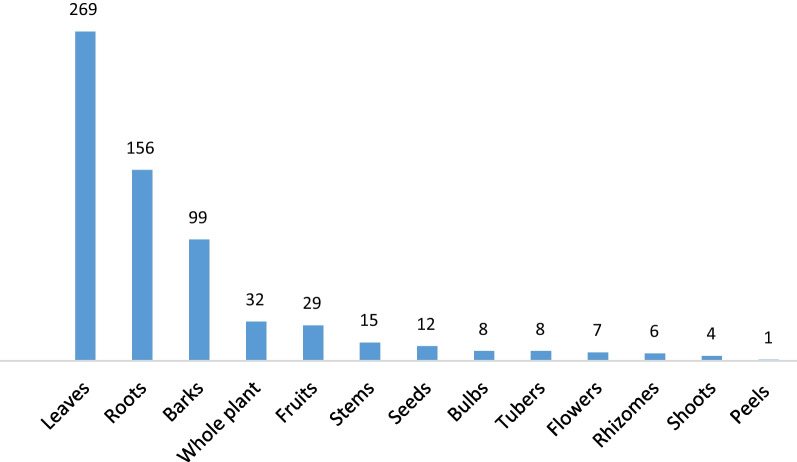
Frequency of the reported plant parts used for herbal preparations

Overall, 94% (*n* = 63) of the included studies mentioned the type of extract/preparation of plant used as an intervention. The most frequent parts of the plants tested were the leaves, roots, barks, whole plant, and fruits (Fig. 3). The findings agree with those from Tanzania [33], Uganda [34], and South Africa [35]. Some rare medicinal plants are vulnerable to extinction and using leaves instead of roots and the whole plant can help preserve them in the long run [36]. This promotes the regular and safe use of leaves in herbal preparations [37]. Traditional healers sometimes prefer roots and bark over leaves because they are easier to store and transport [38]. Hence, it is imperative to apply proper harvesting strategies and conservation measures to ensure sustainable harvesting. This can be done by training traditional healers, herbalists, and others involved in harvesting medicinal plant, to use methods that are less damaging to plants [1, 32]

Additionally, leaves often contain a high concentration of bioactive compounds such as alkaloids, flavonoids, phenolics, and essential oils. These compounds possess medicinal properties and contribute to the therapeutic effects of plants. Traditional healers have recognized the medicinal potential of these compounds and utilise leaves for their healing properties [39, 40].

The routes frequently employed to administer medications during treatment of diarrhea reported in ethnobotanical studies consisted of the following: oral 75% (*n* = 30), nasal 10% (*n* = 4), topical 2.5% (*n* = 1) route of administration, while 12.5% (*n* = 5) did not explicitly state the route of administration used. The results of this study are similar to those reported from Kenya [41], and Ethiopia [42], which also demonstrated that large proportions of medicinal plant remedies were taken orally. The reason for this could be that traditional medicine practitioners choose simple methods, such as oral and topical, to administer treatments, since other administration routes, such as intramuscular and intravenous, require advanced skill [43]. Moreover, as most studies were conducted with ethno-directed plant extracts, it was not plausible to adopt a route other than oral (e.g., intramuscular, intraperitoneal, etc.), which is the typical delivery route for non-fractionated extracts [44]. In contrast, other studies conducted in Southwest Ethiopia, involving the Sheko ethnic group, indicate that medicinal plant remedies were mostly administered topically [45].

**Fig. 4:**
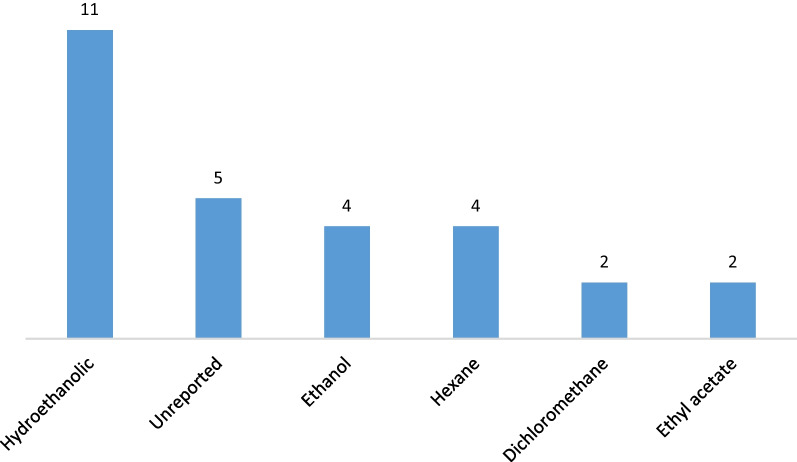
Frequency solvents used for plant extracts

Water, methanol, and acetone were the most used solvents for plant extraction (Fig. 4). This is probably because they allow the extraction of a wide range of active principles without causing toxic side effects. Water was the most used solvent for plant preparation in ethnobotanical studies. This is because, water is an easily accessible, reasonably priced solvent that can dissolve a significant number of metabolites, and high temperatures would enable a quick extraction of active components [46, 47]. The best solvent for extraction relies on the specific plant materials and the compounds that are to be extracted due to the range of bioactive chemicals found in plant materials and their varying solubility qualities in different solvents [46].

**Fig. 5:**
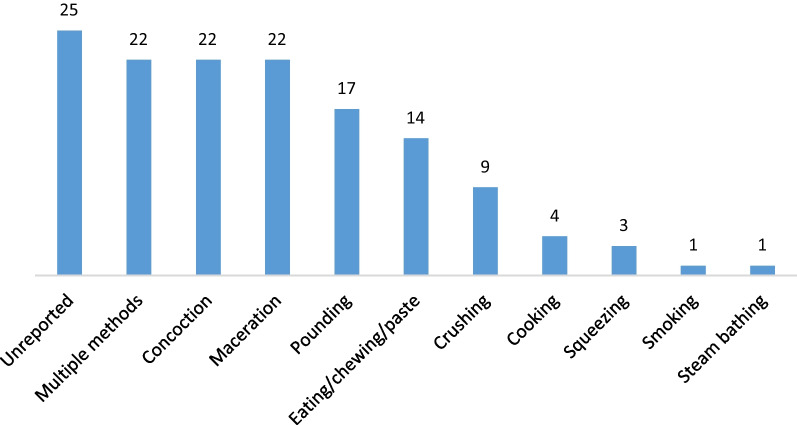
Frequency of herbal preparation and application methods

Traditional healers used either a single or a combination of methods for preparing antidiarrheal herbal remedies. Decoction (*n* = 150), infusion (*n* = 61), maceration and concoction (*n* = 22), and pounding (*n* = 17) represented the most common independent herbal remedy preparations (Fig. 5). In other parts of Africa (Congo, Cameroon, and Kenya), decoction has also been reported as the most commonly used method of preparing herbal remedies [48–50]. As opposed to cold extraction, boiling (decoction) is known to enable the extraction and preservation of herbal medicines for a longer period of time [34]. According to Daswani et al. [51] the usage of plant decoctions at home level can have several drawbacks, such as concerns about changes in efficacy and toxicity profiles with alternative methods, emphasizing the necessity for rigorous testing before advocating for their use. Additionally, the study acknowledges challenges, such as distribution and availability issues, particularly when considering a single-dose formulation from standardized plant material, akin to challenges faced with allopathic drugs. Additionally, Thakkur [52], describes, a typical method for preparing decoction by cooking the plant material until the original volume is reduced to one-fourth. Alternative techniques could include chewing plant material, heating at a lower temperature (like 60 °C), or cold infusion. If these procedures are to be used, they must first be tested before being recommended because any modification to the advised method of preparation may affect its efficacy and/or toxicity profile.

In general, herbal preparations were reportedly given at doses ranging from 5 ml (teaspoon) in children to up to 500 ml a day in adults (Additional file 1: Table S3). In terms of duration, the extracts and preparations were administered only for a short duration of three to five days (until diarrhea stops) in ethnobotanical studies.

A longer duration of consumption of extract, was reported in one study whereby the extract significantly (*P* < 0.01) decreased stool *Shigella* density in diarrheic rats, from the first to the seventh day of treatment in an in vivo study [53].

#### Phytochemistry

Among the 27 included experimental studies (in vitro and in vivo), only ten of them investigated phytochemical analysis (Table 3). It is somewhat problematic that phytochemical characterization studies are limited in SSA countries since they are crucial to the discovery of new therapeutic compounds [54].

**Table 3:** Phytochemical screening for medicinal plants with antidiarrheal activity

Plant species	Solvent	Plant part(s)	Alkaloids	Flavonoids	Tannins	Terpenoids	Saponins	Phenolic compounds	Cardiac glycoside	Anthraquinones	Steroids	Author (year)
*Nymphaea* *lotus* Linn	Methanol	Rhizome	a	a	a	a	a	b	a	a	a	[75]
*Artocarpus* *heterophyllus*	Ethanol and hexane	Seeds	a	a	a	–	a	b	a	–	a	[124]
*Maytenus* *peduncularis,* *Maytenus* *procumbens,* *Maytenus* *senegalensis* and *Maytenus* *undata*	Various solvents	Leaves	b	a	a	b	b	a	b	b	b	[125]
*Indigofera* *lupatana* Baker F	Ethanol	Leaves	b	a	a	a	a	a	a	b	a	[126]
*Dalbergiella* *nyasae*	ethanol	Leaves, roots bark	a	a	a	a	a	a	b	b	a	[96]
*Acacia* *nilotica* (L.) Del., *Acacia* *ethaaica* Schweinf., *Acacia* *tortilis* *(Forssk.)* Hayne., *Cissus* *quadrangularis* L., *Clerodendrum* *myriacoides*	Methanol	Bark/roots Bark Roots Stems Roots	a	a	a	a	a	b	a	b	b	[60]
*Acacia* *horrida* (L.) Willd	Methanol	Bark	a	–	a	a	a	b	a	b	b	[60]
*Acacia* *nubica* Benth	Methanol	Bark	–	a	–	a	–	b	a	b	b	[60]
*Acacia* *senegal* (L.)	Methanol	Bark	–	a	a	a	a	b	a	b	b	[60]
*Acokanthera* *friesiorum* Markgr	Methanol	Roots/leaves	–	a	a	–	–	b	a	b	b	[60]
*Albizia* *anthelmitica* Brongn	Methanol	Roots/leaves	a	a	a	–	a	b	–	b	b	[60]
*Aloe* *secundiflora* Engl	Methanol	Whole	–	a	a	a	–	b	–	b	b	[60]
*Balanites* *aegyptiaca* (L.) Del	Methanol	Roots	a	a	a	a	a	b	–	b	b	[60]
*Boscia* *angustifolia* Guill. and Perr	Methanol	Bark	a	–	a	–	–	b	–	b	b	[60]
*Cissus* *rotundifolia* *Forsk.* *Vahl*	Methanol	Roots	a	–	a	–	a	b	–	b	b	[60]
*Cordia* *monoica* Roxb	Methanol	Roots	–	–	a	a	a	b	–	b	b	[60]
*Anacardium* *occidentale* L	Aqueous, hydroethanolic	Leaves	–	–	a	–	a	b	b	b	–	[73]
*Daniellia* *oliveri* *(Rolfe)* Hutch. *&* Dalziel	Aqueous, hydroethanolic	Leaves	a	a	a	–	a	b	b	b	–	[73]
*Diospyros* *mespiliformis* *Hochst.* ex A. DC	Aqueous, hydroethanolic	Leaves	a	a	a	–	a	b	b	b	–	[73]
*Khaya* *senegalensis* *(Desr.)* A. Juss	Aqueous, hydroethanolic	Bark	a	a	a	–	a	b	b	b	–	[73]
*Manihot* *esculenta* Crantz	Aqueous, hydroethanolic	Leaves	–	–	a	–	a	b	b	b	–	[73]
*Ocimum* *gratissimum* L	Aqueous, hydroethanolic	Leaves	a	a	a	–	a	b	b	b	–	[73]
*Pterocarpus* *erinaceus* Poir	Aqueous, hydroethanolic	Leaves	–	a	a	a	–	b	b	b	a	[73]
*Rauvolfia* *vomitoria* Afzel	Aqueous, hydroethanolic	Leaves	–	a	a	a	a	b	b	b	a	[73]
*Senna* *italica* Mill	Aqueous, hydroethanolic	Leaves	–	–	a	–	a	b	b	b	–	[73]
*Vernonia* *amygdalina* *D*	Aqueous, hydroethanolic	Leaves	a	a	a	a	a	b	b	b	a	[73]
*Priva* *adhaerens*	Aqueous	Leaves, shoot	a	–	a	b	a	b	b	–	b	[74]
*Osyris* *quadripartita* *D*	Methanol	Leaves	a	a	a	a	a	a	–	a	–	[127]
*Hydnora* *abyssinia* *A.Br.* *a* *Harrisonia* *abyssinica* *Oliv.b*	Ethanl, ethyl acetate	Roots, stems	a	a	a	a	a	b	b	b	–	[128]
*Solanum* *incanum* *L*	Ethanl, ethyl acetate	Roots	a	a	a	a	a	a	a	a	a	[128]
*Leucas* *aspera* *(Willd.)* *Link*	Ethanl, ethyl acetate	Leaves	–	a	a	a	a	b	b	b	–	[128]

^a^Present

^b^Not done

– Absent

Six compounds were isolated and identified with anti-diarrheal activity from *Hydnora* *johannis* as follows: cirsiliol (3′,4′,5-trihydroxy-6–7-dimethoxy flavone), trans 3′5-dihydroxy-4′7-dimethoxydihydroflavonol, oleic acid, vanillin (4-hydroxy-3-methoxybenzaldehyde), protocatechuic acid (3,4-dihydroxy benzoic acid) and DL catechin (*trans* (+) 2-(3,4-dihydroxyphenyl-3,4-dihydro-2*H*-1-benzopyran-3,5,7-triol)) [55]. Some of the secondary metabolites screened were flavonoids, saponins, tannins, alkaloids, and terpenoids. The species of the most common families (*Asteraceae* and *Fabaceae*) of medicinal plants used for diarrhea treatment in the current study are known to contain a variety of phytochemicals, including flavonoids, saponins, alkaloids, lectins, phenolic acids, and carotenoids [17, 56]. According to Otshudi et al. [57] and Maroyi, [58], these active ingredients have been linked to their ability to prevent or treat ailments, such as diarrhea. Flavonoids are believed to have anti-diarrheal effects by inhibiting intestinal motility and hydro-electrolytic secretion, which are altered in this intestinal condition [59]. Tannins were detected in all plant species, except for *Acacia* *nubica* [60]. They are known for their astringent and anti-inflammatory properties [61]. Studies have shown that tannins have anti-diarrheal effects through inhibition of intestinal motility and hydroelectrolyte secretion [62, 63].

Bisi-Johnson et al. [64] employed Thin layer chromatography (TLC) plates as a technique to separate the extracts into their constituent chemicals. The analysis revealed the presence of terpenoids and flavonoids in the herbs. In previous studies, plant species possessing both terpenoids and flavonoids demonstrated anti-diarrheal activity [65, 66]. Analyzing the phytochemical composition of plant preparations and identifying their active components is helpful in understanding anti-diarrheal mechanisms [67]. Such information allows the discovery of molecules with the biotechnological potential to be used in the development of new medicines [68].

#### Toxicology (safety findings)

In traditional medicine settings, medicinal plants ascribe their pharmacological effect to their active and ‘safe’ content which only exerts a quick effect when taken in large doses [32]. Toxicity to the host was reported in 13 studies (40.7%); the median lethal dose (LD_50_) test (54.5%) was the most widely used.

The few investigations on the toxicology of medicinal plants highlight the measures that must be taken before integrating traditional medicine into standard treatment [69]. For example, several therapeutic herbs may include mutagenic and carcinogenic compounds, which may manifest their effects over time [70]. In the present scoping review, this aspect was identified by some studies. Three plants, *Elaeodendron* *croceum,* *Calpurnia* *aurea,* and *Maesa* *lanceolata,* showed relatively low cytotoxicity with LC50 > 20 μg/ml against Vero monkey kidney cells [71]. In another study, at a concentration of 10 μg/ml the water and ethanol extracts of *Hydnora* *johannis* showed moderate toxicity on KB cells lines with 41% ± 5 and 65% ± 3, respectively [55]. Despite being poorly understood, host toxicity may be caused by the highly concentrated extracts used in the treatments, which are very different from ethnomedicine, which is primarily based on less concentrated plant extracts [44].

Plant extracts from *Dodonaea* *viscosa,* *Khaya* *senegalensis,* *Daniellia* *oliveri,* *Rauvolfia* *vomitoria,* *Vernonia* *amygdalina,* *Manihot* *esculenta,* *Ocimum* *gratissimum,* *Senna* *italica,* *Diospyros* *mespiliformis,* *Pterocarpus* *erinaceus,* and *Anacardium* *occidentale,* reported LD_50_ greater than 2000 mg/kg in Swiss albino mice and Wistar albino rats, respectively [72, 73]. Other plants (*Nymphaea* *lotus* and *Priva* *adhaeren*s) showed oral LD_50_ greater than 5000 mg/kg body weight in Swiss albino mice [74, 75]. Based on Lorke’s chemical classification, these extracts are non-toxic when consumed orally [76]. In this group of animals (both rats and mice), this dose did not alter behaviour or cause any deaths. Therefore, both plant species *(Nymphaea* *lotus* and *Priva* *adhaeren*s) extracts were considered to be safe at doses ≤ 5000 mg/kg. Additionally, we found that castor oil-induced diarrhea in rats was significantly inhibited by the extracts, comparable to that of loperamide, the standard anti-diarrheal drug. There is no evidence that the plant products tested are safe since more than half of the studies included in this review did not test their host toxicity. This presents a serious problem since positive results cannot be considered alone to assess the biological/pharmacological relevance of the tested product, a risk–benefit analysis must be analysed to determine its biological/pharmacological relevance [77, 78]. Despite the credit ethnobotany has received in drug discovery, consumers should be cautious when using traditional knowledge of medicinal plants as not all traditional knowledge has been therapeutically tested [79].

#### Ethnobotanical findings

Ethnobotany is a branch of science that focuses on the traditional uses of plants, such as their usage as medicines [80]. The ethnobotanical data is frequently used to choose efficacy trials for medicinal plants. This type of valuable information is largely founded on years of beliefs and experience, and it is more plentiful in nations with significant ethnic diversity since more indigenous tribes there have used or tried alternative medical treatments like employing plants to treat illnesses [80, 81]. Ethnobotanical studies require standard procedures for botanical identification, as well as reliable documentation of indigenous knowledge regarding plant management, distribution, and traditional use [32]. Forty (*n* = 40) ethnobotanical studies were included in this review which are all collective survey studies that involved 3 523 local residents (the majority were traditional health practitioners) in the studied countries (Additional file 1: Table S3). The studies were conducted in 18 SSA countries mostly in Ethiopia (*n* = 8), followed by South Africa (7), Mozambique, Mauritius, and Kenya (*n* = 3), respectively. Other countries include Nigeria, Lesotho, Congo, Sudan, Swaziland, Cameroon, Madagascar, Zimbabwe, Tanzania, Mali, Ghana, Uganda, and Angola. However, certain information could not be found in nine articles (22.5%), such as herbal preparation and application route.

*Elephantorrhiza* *elephantina* is the only plant that was analysed in more than one study. This herb is used in southern Africa as a traditional remedy for many ailments and diseases, including digestive disorders, dermatological disorders, wounds, sexually transmitted infections, and sexual dysfunction. Its multifaceted traditional role, coupled with a scarcity of alternative remedies in the region, likely spurred researchers to delve deeper into its therapeutic potential. Moreover, if initial studies yielded promising results regarding its efficacy and safety, this could have catalyzed a cascade of subsequent investigations. *Elephantorrhiza* *elephantina’s* distinct pharmacological profile and cultural significance may have further fueled scientific curiosity, positioning it as a focal point for sustained research efforts aimed at unraveling its comprehensive healing properties and validating its traditional uses [82].

#### In vitro findings

Overall, most in vitro studies 63% (*n* = 7) reported good activity, while 27% (*n* = 3) reported moderate activity. All included in vitro studies tested the antimicrobial activities of medicinal plants against diarrhea-causing agents.

In the current study, several plant species showed substantial variation in anti-diarrheal activity between studies. For instance, there was significant variation in antidiarheal activity in some plant species including: *Indigofera* *lupatana,* *Hydnora* *johannis,* *Psidium* *guajava,* *Dalbergiella* *nyasae,* *Lippia* *javanica,* *Canarium* *schweinfurthii,* *Senna* *occidentalis,* *Vernonia* *natalensis,* *Cyathula* *uncinulata*, *Syzygium* *cordatum,* *Isoglosa* *lacteal,* and *Gymnosporia* *senegalensis.* It is possible that these differences were caused by differences in the extraction solvent, resulting in different extraction yields and extracted secondary metabolites. Ethanol was the most used solvent, probably because it has a higher polarity than most non-polar compounds, but a lower polarity than water. Ethanol with 96% concentration can pass through and penetrate cells very easily and achieve higher-concentration extractions [83]. Other solvents used were methanol, acetone, hexane, dichloromethane, ethyl acetate, and water, in that order.

The studies mostly investigated crude extracts (72.7%), and rarely the isolated (pure) compounds (9.1%). One study investigated both crude extracts and fractions (9.1%), and another, only fractions (9.1%). Most studies in SSA countries used only crude extracts of plants, possibly due to the lack of infrastructure needed to process the materials into pure compounds. Moreover, geographical differences may affect the activity of the same plant species depending on where they were collected [84]. A suitable solvent for extracting the target compound from the plant material is also crucial [85]. For example, a study conducted in KwaZulu-Natal, South Africa, found *P.* *guajava* to have poor anti-diarrheal activity [86], while another study with the same plant species in the Eastern Cape, South Africa, revealed a good anti-diarrheal activity [64]. It is important to note, however, that the extraction solvents used in both studies were different.

The lowest minimum inhibitory concentration (MIC) value was 0.039 mg/ml for the majority of the tested diarrhea-causing bacteria (*Staphylococcus* *aureus,* *Vibro* *cholera,* *Shigella* *dysentery*, and *S.* *flexneri*); in ethanol and acetone crude extracts of *Indigofera* *daleoides* and *Punica* *granatum*. From the MIC results obtained in the present, it can be concluded that both plants, could be a good source of bioactive components with antimicrobial potency [87]. The other plant with antimicrobial activities was *Harrisonia* *abyssinica*, in which ethyl acetate extracts had MIC of < 625 μg/ml and ethanol extracts with MIC values of 625–1250 μg/ml against all the tested microorganisms (*S.* *aureus,* *E.* *feacalis,* *E.* *coli,* and *S.* *typhimurium*). The results of this study are in agreement with a study by Kareru et al. [88] on the same plant. The in vitro results of this study demonstrated that the stem extract inhibited pathogenic Gram-negative and Gram-positive bacteria, which is consistent with the findings by Cyrus et al. [89] from earlier research on the antibacterial activity of this plant stem. They discovered that the plant stem inhibits *Bacillus* *cereus*, *S.* *aureus,* *P.* *aeruginosa*, and *E.* *coli*.

In this review, it was observed that pure compounds isolated from the ethyl acetate fraction of *Hydnora* *johannis* did not show any activity against *S.* *aureus* [55]. This is in contrast with some studies [90, 91] which have reported that activities of the medicinal plants, used to treat infectious diseases, increased with the isolation of the active compounds, thus confirming the necessity of research to identify the active compounds of SSA medicinal plants. For example, an in vitro study [92] showed quercetin; a major flavonoid present in *P.* *guajava* leaves, had significant anti-diarrheal effects on guinea pig ileum contraction and mouse small intestine contraction as well as reduced abdominal capillary permeability [93].

The aqueous extracts from *T.* *sericea* were the only plant species among the other 22 to exhibit notable efficacy against five of the seven pathogens (*B.* *cereus,* *E.* *faecalis,* *P.* *vulgarius,* *S.* *typhimurium*, *S.* *aureus,* *E.* *coli,* and *S.* *flexneri*) under investigation [87]. The medicinal properties of *T.* *sericea*, one of the most significant plants used in traditional African medicine, have been shown to exhibit marked anti-fungal, anti-HIV, anti-cancer, anti-bacterial, anti-inflammatory, lipolytic, antiparasitic, wound-healing, and anti-oxidant action [94]. Two (18.2%) of the eleven in vitro studies determined minimum fungicidal concentration (MFC). In one study, the lowest MFC value of 160 μg/ml from *Dalbergiella* *nyasae* was recorded against one yeast species (*Candida* *albicans*). In this study, yeast was found to be the most susceptible organism after Gram-positive bacteria *S.* *aureus* and Gram-negative *E.* *coli* and *P.* *aeruginosa*. Moreover, minimum bactericidal concentration (MBC) was also determined, which is the lowest concentration at which an antimicrobial agent will kill a particular microorganism. MFC/MBC (0.625–5 mg/ml, 0.625–10 mg/ml, 0.625–10 mg/ml, and 0.625–2.5 mg/ml) values against *P.* *aeruginosa*, *S.* *aureus,* *E.* *coli,* and *C.* *albicans* respectively, were higher than the MIC (0.31–2.5 mg/ml, 0.31–5 mg/ml, 0.16–5 mg/ml, and 0.16–1.25 mg/ml) values of the extract, indicating that the extracts had a bacteriostatic/fungistatic effect on the tested microorganisms [95]. However, in another study, dichloromethane, ethyl acetate and ethanol extracts of five plants were tested for antimicrobial activity, and it was reported that all the extracts were inactive against the two fungi (*C.* *albicans* and *Cryptococcus* *neoformans*) used, except the dichloromethane extract of *Whitfieldia* *elongate* which exhibited strong antifungal activity against *C.* *neoformans*. Although some extracts may not possess very strong activities, it is worth mentioning that some of the plants are used in combination to enhance their efficacy with the combination of *Dissotis* *brazzae* and *Solanum* *nigrum* (Solanaceae) as an example [96].

#### In vivo findings

Only one study did not describe the animal lineage (14.3%), and those reporting this parameter used Wistar rats 42.9% (*n* = 3), Swiss mice 28.6% (*n* = 2) or New Zealand rabbits 14.3% (*n* = 1). All studies described the sex of the animals. The weight of the animals ranged from 20 to 30 g in mice, 100–300 g in rats, and 1.5 kg average for rabbits. The age ranges from 6 to 8 weeks for mice, and three months for rats. However, this variable was not reported by most studies 71.4% (*n* = 5). Guidelines for reporting animal experiments require that the body weight and age of the animals be stated [97, 98]. But since this variable was neglected in the majority of studies, it can be difficult to convert animal data to humans because, despite being available, these data are typically not disclosed [99–101]. The age and body weight of animals can have an impact on gene expression, metabolic parameters, drug metabolism, and other dependent variables that are assessed in animal experiments [102]. Having this information on hand would allow comparisons between studies and bring an understanding, of what role immune responses may have played in the results of different treatments investigated.

In the vast majority of the experiments, 57.1% (*n* = 4) reported good activity, and 42.9% (*n* = 3) reported moderate activity (Additional file 1: Fig. S1). Plant families reported for the in vivo studies were Asteraceae (*n* = 2), Leguminosae (*n* = 2), Lamiaceae (*n* = 2), Euphorbiaceae (*n* = 2), Verbenaceae (*n* = 2), Sapindaceae, Santalaceae, Myrtaceae, Mimosaceae, Fabaceae, Anacardiaceae, Ebenaceae, Meliaceae, Apocynaceae, and Rutaceae. The Asteraceae family, which has a widespread distribution and is the second-largest Angiosperm family, is well known for having a number of phytochemical qualities [103].

Different animal models have been utilized including rats 42.9% (*n* = 3), mice 28.6% (*n* = 2), both (rats and mice) 14.3% (*n* = 1), and both (rats and rabbits) 14.3% (*n* = 1). Rats are preferred animal models due to their similar physiological, anatomical, and genetic characteristics to humans. Mice are also referred to as useful animal models [104]. In the study by Bello et al. [75], rabbits were deliberately included to diversify the study’s scope and better understand the potential effects of the methanol rhizome extract of *Nymphaea* *lotus* on a range of species. The unique physiological traits of rabbits offer complementary perspectives to the findings derived from rat and mouse models, enriching the overall comprehension of the extract’s pharmacological impact across different organisms.

The most widely used anti-diarrheal drug as a positive control for in vivo studies includes loperamide 85.7% (*n* = 6) (Additional file 1: Table S2). Loperamide is classified as an anti-diarrheal agent. Loperamide was approved by the Federal Drug Administration to treat various forms of diarrhea, including irritable bowel syndrome associated with chronic diarrhea, and acute nonspecific diarrhea in adults and children over the age of two [105].

There was a significant difference in the duration of treatment among in vivo studies, ranging from 30 min to 14 days. Oral administration was the preferred route of administration for treatment (*n* = 6), while another route describe was intragastric (*n* = 1). The oral route is the most preferred route for drug administration due to its advantages, including non-invasiveness, and convenience of use. There are many factors that affect oral drug absorption, including drug solubility, and gastrointestinal tract stability [106].

In another study by Wambe et al. [53], water/ethanol *Cola* *anomala* pods extract demonstrated bactericidal activity, with a MIC of 2.0 mg/ml. This plant obtained the most promising in vivo outcomes with decreased stool *Shigella* density and significantly (*P* < 0.01) raised white blood cells in diarrhoeic rats. As a result of the treatment, some damage was repaired to the eroded epithelium of the intestine, weight loss was prevented, and nitric oxide, IL-1β, and TNF-α levels fell significantly in the colon. Bioactive substances in *C.* *anomala* extract may directly affect the destruction of *S.* *flexneri*. Furthermore, it was reported that *Cola* species contains alkaloids and phenolic compounds, where phenolic compounds are implicated in the rupture of the membrane of bacteria, thereby increasing their permeability [107].

#### Cross-sectional studies

Two cross-sectional studies were included in this review. Mwambete and Joseph [108] interviewed a total of 161 mothers, of those, 74 (46%) were female and 87 (54%) were male under-fives with a median age of 2 years. The most widely used remedy for the treatment of diarrhea was *Psidium* *guayava* 28% (*n* = 45), locally known as Mpera (Tanzania). Globally, the plant is used to treat diarrhea, dysentery, cough, fever, malaria, ulcers, boils and wounds, indigestion gastroenteritis, stomachaches, and inflammation [109–111]. It is also known that several chemical compounds isolated from guava leaves possess antibacterial properties against strains of gram-positive bacteria [112] and gram-negative bacteria [113].

According to Mwambete and Joseph [108], three-fifths of respondents cited metronidazole (flagyl) and oral rehydration solution (ORS) are the most effective conventional chemotherapeutic drugs that are often used to treat diarrheal issues. As additional effective treatments for diarrhea, wheat flour, a solution of water and ash and were also recommended.

## Summary of major findings

This scoping review systematically synthesizes the wealth of information regarding the use of medicinal plants for treating diarrhea in Sub-Saharan Africa (SSA). Over the past two decades, a notable surge in research on medicinal plants has been observed, with a particular uptick since 2008. South Africa, Ethiopia, and Uganda emerged as significant contributors, driven by their robust investments in research and development. These countries, known for their deep-rooted reliance on traditional medicine, demonstrated heightened scholarly outputs, underscoring a strong correlation between cultural practices and research endeavors. The dominance of specific plant families, including Fabaceae and Asteraceae, underscores the botanical richness characterizing SSA. Noteworthy plant species, such as *Psidium* *guajava*, *Syzygium* *cordatum*, and *Vernonia* *amygdalina*, have garnered substantial research attention, unveiling a correlation between research intensity and the therapeutic potential of certain plants.

In exploring the multifaceted landscape of interventions, diverse plant parts such as leaves, roots, barks, fruits have been investigated, each offering unique bioactive compounds. Ethnobotanical insights from 40 collective surveys across 18 SSA countries shed light on the traditional uses of medicinal plants, showcasing the intricate interplay between indigenous knowledge and therapeutic practices Phytochemical analyses uncovered active compounds such as flavonoids, saponins, and alkaloids, elucidating the mechanisms behind the anti-diarrheal properties of these plants. Noteworthy findings include the isolation of six compounds with anti-diarrheal activity from *Hydnora* *johannis* and the varied antimicrobial efficacy observed across plant species, such as *Indigofera* *daleoides* and *Punica* *granatum*. However, safety concerns were unveiled through toxicology assessments, with LD_50_ tests revealing potential risks in approximately 40% of the studies. Cross-sectional studies further enriched the understanding of local practices and preferences, adding a contextual layer to the broader narrative.

## Strength, limitations, and future study direction

This scoping review provided valuable insights into the efficacy and safety of some important anti-diarrheal medicinal plants in SSA. It further, demonstrated that most local people of SSA are familiar with the uses of medicinal plants and that they frequently turn to traditional remedies to treat diarrheal illnesses. Although systematic review and meta-analysis are the more rigorous criteria and preferred methods for performing reviews, they are not the exclusive. Scoping reviews, which are typically used to highlight knowledge gaps, are allowed.

While presenting the findings of this review, it is crucial to acknowledge several limitations. One notable concern revolves around the selection of informants participating in Sub-Saharan Africa (SSA) ethnobotanical investigations. The number of informants chosen varies widely across studies, ranging from 4 to 38 [58, 115–121], impacting the generalizability of the conclusions drawn on the research subject. The small sample sizes raise questions about whether these numbers accurately represent the entire spectrum of indigenous knowledge within a given district. As highlighted by Pananiagua-Zambrana et al. [122], ethnobotanical studies benefit significantly from indigenous interviewers and a diverse range of participants. Considering the diverse knowledge held by individuals and the time constraints faced by external investigators, employing multiple local interviewers appears to be the optimal approach for conducting ethnobotanical studies.

Furthermore, no clinical trials have been conducted using plants with potential anti-diarrheal activity. Also, most of the studies did not report the composition of the formulation, standardization protocols, and preparation procedures. This limitation was also observed in a review by Maroyi [69] on a similar subject matter. The study emphasised the significance of conducting clinical studies on these selected species in order to establish the optimal dosages and formulations as well as, to evaluate the effects in humans.

This current review also discovered that only a small number of active compounds have been identified and tested for effectiveness against diarrheal diseases. Plants having low MIC levels in their extracts should undergo this isolation and pharmacological examination. In this work, we identified only one plant species, i.e., *H.* *johannis*. Although pure chemical products or compounds from medicinal plants with higher antibacterial activity have been isolated, elucidating their biological mechanisms and conducting pharmacological studies remains a major challenge [122].

Additionally, it is important to note that while bacterial enteropathogens (i.e., *S.* *aureus,* *E.* *coli,* *S.* *typhimurium,* *P.* *aeruginosa,* and *E.* *faecalis)*, tested in the included studies are frequently associated with diarrhea, other pathogens such as rotaviruses and parasites such as *Entamoeba* *histolytica* may also contribute to the burden of diarrhea. Considering this, further research on these neglected pathogens should be conducted.

The review findings suggest that there are significant knowledge gaps about the possible toxicity of herbs in SSA, when evaluating their performance efficiency as anti-diarrheal agents. A significant barrier to the efficient development and use of local medicinal plant resources is represented by this gap [32]. Standardizing (the preparation, dosage, and route of administration) and authenticating plant species that have anti-diarrheal properties can mitigate this challenge [123]. These initiatives will aid in the development of medicines that are efficient in treating different diarrheal illnesses. Researchers can use this data to target future phytochemical and pharmacological investigations on these medicinal plants.

## Conclusion

In conclusion, this scoping review highlighted the extensive use of medicinal plants in Sub-Saharan Africa (SSA) for treating diarrhea, revealing a rich landscape of anti-diarrheal potential. However, significant knowledge gaps were identified, underscoring the necessity for further in vitro*,* in vivo, ethnobotanical, and cross-sectional studies to strengthen evidence on the therapeutic efficacy of commonly used medicinal plants in SSA. The recognized lack of information in specific areas guides future research efforts. Health professionals, researchers, and consumers can use these insights to navigate guidelines, inform decision-making, and plan more effective research. Recommendations include health professionals exercising caution and collaborating for precise dosage guidelines, researchers addressing knowledge gaps through targeted studies and community engagement, and consumers approaching traditional medicinal plant use with awareness, seeking professional advice, and participating in community education programs. The multidisciplinary approach in clinical research remains vital to establish effective doses, mitigate potential harm, and ensure the safety of anti-diarrheal agents from medicinal plants in SSA.

### Supplementary Information

**Additional file 1: Table S1: **Preferred Reporting Items for Systematic reviews and Meta-Analyses extension for Scoping Reviews (PRISMA-ScR) Checklist. **Table S2** Summary of included in vitro, in vivo and cross-sectional studies using medicinal plants as antidiarrheal treatment. **Table S3**: Summary of included ethnobotanical information studies using medicinal plants as antidiarrhoeal treatment. **Figure S1: **Summary of included studies reporting the plant species with good, moderate, and/or least activity for diarrhoea treatment.

## Data Availability

Data will be made available on request.
