# Supplementary material for: A scoping review on efficacy and safety of medicinal plants used for the treatment of diarrhea in sub-Saharan Africa
Source: Trop Med Health. 2024 Jan 3;52:6. doi: 10.1186/s41182-023-00569-x (PMC10763068; doi:10.1186/s41182-023-00569-x)
Supplement: Supplementary file 1 — Additional file 1: Table S1: Preferred Reporting Items for Systematic reviews and Meta-Analyses extension for Scoping Reviews (PRISMA-ScR) Checklist. Table S2 Summary of included in vitro, in vivo and cross-sectional studies using medicinal plants as antidiarrheal treatment. Table S3: Summary of included ethnobotanical information studies using medicinal plants as antidiarrhoeal treatment. Figure S1: Summary of included studies reporting the plant species with good, moderate, and/or least activity for diarrhoea treatment. [file 41182_2023_569_MOESM1_ESM.docx]

**A scoping review on efficacy and safety of medicinal plants used for the treatment of diarrhea in sub-Saharan Africa**

**Moitshepi T.A. Plaatjie^1^, ThankGod E. Onyiche^2^,** [**Tsepo Ramatla**](https://www.researchgate.net/profile/Tsepo-Ramatla)**,^1,5*^, Johannes J. Bezuidenhout^1^, Lesetja Legoabe^3^, Nthatisi I. Nyembe^4^ and Oriel Thekisoe^1^**

^1^ Unit for Environmental Sciences and Management, North-West University, Potchefstroom, South Africa

^2^ Department of Veterinary Parasitology and Entomology, University of Maiduguri, Maiduguri 600230, Nigeria

^3^ Pharmaceutical Chemistry, School of Pharmacy, North-West University, Potchefstroom 2520, South Africa

^4^ Department of Zoology and Entomology, University of the Free State, Phuthaditjhaba, South Africa

^5^ Gastrointestinal Research Unit, Department of Surgery, School of Clinical Medicine, University of the Free State, Bloemfontein 9300, South Africa

*Corresponding author: Tsepo Ramatla

E-mail addresses: [ra21205450@gmail.com](mailto:ra21205450@gmail.com), Tel: +27-18-299-2521

**Supplementary materials**

**Supplementary Table S1:** Preferred Reporting Items for Systematic reviews and Meta-Analyses extension for Scoping Reviews (PRISMA-ScR) Checklist

| **SECTION** | **ITEM** | **PRISMA-ScR CHECKLIST ITEM** | **REPORTED ON PAGE #** |
| --- | --- | --- | --- |
| TITLE | | | |
| Title | 1 | Identify the report as a scoping review. | 1 |
| ABSTRACT | | | |
| Structured summary | 2 | Provide a structured summary that includes (as applicable): background, objectives, eligibility criteria, sources of evidence, charting methods, results, and conclusions that relate to the review questions and objectives. | 1-2 |
| INTRODUCTION | | | |
| Rationale | 3 | Describe the rationale for the review in the context of what is already known. Explain why the review questions/objectives lend themselves to a scoping review approach. | 4 |
| Objectives | 4 | Provide an explicit statement of the questions and objectives being addressed with reference to their key elements (e.g., population or participants, concepts, and context) or other relevant key elements used to conceptualize the review questions and/or objectives. | 4-5 |
| METHODS | | | |
| Protocol and registration | 5 | Indicate whether a review protocol exists; state if and where it can be accessed (e.g., a Web address); and if available, provide registration information, including the registration number. | 4 |
| Eligibility criteria | 6 | Specify characteristics of the sources of evidence used as eligibility criteria (e.g., years considered, language, and publication status), and provide a rationale. | 5 |
| Information sources* | 7 | Describe all information sources in the search (e.g., databases with dates of coverage and contact with authors to identify additional sources), as well as the date the most recent search was executed. | 5 |
| Search | 8 | Present the full electronic search strategy for at least 1 database, including any limits used, such that it could be repeated. | 42, Table 1 |
| Selection of sources of evidence† | 9 | State the process for selecting sources of evidence (i.e., screening and eligibility) included in the scoping review. | N/A |
| Data charting process‡ | 10 | Describe the methods of charting data from the included sources of evidence (e.g., calibrated forms or forms that have been tested by the team before their use, and whether data charting was done independently or in duplicate) and any processes for obtaining and confirming data from investigators. | 6-7 |
| Data items | 11 | List and define all variables for which data were sought and any assumptions and simplifications made. | 5 |
| Critical appraisal of individual sources of evidence§ | 12 | If done, provide a rationale for conducting a critical appraisal of included sources of evidence; describe the methods used and how this information was used in any data synthesis (if appropriate). | 6-7 |
| Synthesis of results | 13 | Describe the methods of handling and summarizing the data that were charted. | 6-7 |
| RESULTS | | | |
| Selection of sources of evidence | 14 | Give numbers of sources of evidence screened, assessed for eligibility, and included in the review, with reasons for exclusions at each stage, ideally using a flow diagram. | 39, Fig 1 |
| Characteristics of sources of evidence | 15 | For each source of evidence, present characteristics for which data were charted and provide the citations. | S2 Table, S3 Table |
| Critical appraisal within sources of evidence | 16 | If done, present data on critical appraisal of included sources of evidence (see item 12). | N/A |
| Results of individual sources of evidence | 17 | For each included source of evidence, present the relevant data that were charted that relate to the review questions and objectives. | S2 Table, S3 Table |
| Synthesis of results | 18 | Summarize and/or present the charting results as they relate to the review questions and objectives. | Fig 2, Fig 3, Fig 4, Fig 5, S1 Fig 1, Table 2, Table 3 |
| DISCUSSION | | | |
| Summary of evidence | 19 | Summarize the main results (including an overview of concepts, themes, and types of evidence available), link to the review questions and objectives, and consider the relevance to key groups. | 7-21 |
| Limitations | 20 | Discuss the limitations of the scoping review process. | 21-23 |
| Conclusions | 21 | Provide a general interpretation of the results with respect to the review questions and objectives, as well as potential implications and/or next steps. | 23 |
| FUNDING | | | |
| Funding | 22 | Describe sources of funding for the included sources of evidence, as well as sources of funding for the scoping review. Describe the role of the funders of the scoping review. | 8; 24 |

**Supplementary Table S2** Summary of included *in vitro, in vivo* and cross-sectional studies using medicinal plants as antidiarrheal treatment

| **Study type**  **(Design)** | **Author**  **(Year)** | **Funding** | **Country** | **Experimental model** | **Plant species** | **Intervention details** | **Comparator** | **Main findings** |
| --- | --- | --- | --- | --- | --- | --- | --- | --- |
| 1. Combination (*In vitro* and *in vivo*) | [76] | Not reported | Nigeria | Rabbit jejunum from New Zealand adult male rabbit, and guinea pig ileum (*in vitro*). Ninety adult Swiss albino mice (20–25 g) of both sexes (*in vivo*). | *Nymphaea lotus Linn.* | Dried rhizome powder with 80% aqueous methanol | Loperamide  (3 mg/kg p.o.) | For castor oil-induced diarrhoea, the extract at doses of 200, 400 and 800 mg/kg produced significant reduction in the frequency of diarrhoea (at p<0.001, p<0.001 and p<0.01 respectively). The extract at 800 mg/kg produced a significant delay in onset of diarrhoea (p<0.05) comparable to loperamide (3 mg/kg) |
| 1. Combination *(In vitro* and *in vivo)* | [54] | Not reported | Cameroon | Shigella flexneri (*in vitro*)  Male and female healthy Wistar albino rats (90–200 g, five groups of five animals each) (*in vivo*) | *Cola anomala* | Powder was extracted with a water/ ethanol mixture (V/V). For this, 1000 g of powder was macerated for 72 h | Ciprofloxacin  (2.5 mg/kg) | *In vitro*, water/ethanol *Cola anomala* pods extract showed to be bactericidal, with a minimum inhibitory concentration of 2.0 mg/ml and a minimum bactericidal concentration of 3.0 mg/ml. In diarrheic rats, the extract significantly (P < 0.01) increased the white blood cells and significantly (P < 0.01) decreased stool *Shigella* density from the first to the seventh day of treatment |
| 1. Combination (Ethnobotanical and *in vitro*) | [89] | Public | South Africa | Ethnobotanical: Four traditional healers (two men and two women) were interviews in three localities, Mentz, Botlokwa and Seshego of the Capricon District in Limpopo Province, South Africa.  *In vitro*:  *The bacterial species used, S. aureus* ATCC 25923, *lmonella typhy* ATCC 0232, *V. cholera*, *E. coli* ATCC 35218 and *Shigella* spp. batch 0.57 (*S. dysentery, S. flexneri, S. sonnei, S. boydii*). | *Aloe greatheadii Schonl.*  *Asparagus cooperi* Bak.  *Bidens pilosa L.*  *Bulbine natalensis Bak. cf. roowortel*  *Carpobrotus edulis* (L.) N. E. Br.  *Combretum imberbe Wawra.*  *Elephantorrhiza burkei Benth.*  *Elephantorrhiza elephantina (Burch.) Skeels*  *Guilleminea densa* Moq.  *Gymnosporia senegalensis Loes.*  *Flex mitis* L. Radlk*.*  *Indigofera daleoides Benth. ex Harv. & Sond.*  *Ozoroa insignis Delile.*  *Punica granatum L.*  *Schotia brachypetala Sond.*  *Sclerocarya birrea* (A. Rich)  *Solanum supinum Dun.*  *Spirostachys africana* Sond.  *De wet cordatum Hochst.* ex C. Krauss.  *Waltheria indica L.*  *Ximenia caffra* Sond. Var. caffra | Dried powder, extracted with methanol, acetone, ethanol and boiling water; filtered and freeze dried | Nalidixic acid | The most active extracts were those obtained from Punica granatum and *Indigofera daleoides*. The MIC values for active extracts ranged between 0.039 and 0.6 mg/ml |
| 1. Combination (Ethnobotanical and *in vitro*) | [129] | Not reported | Ethiopia | Ethnobotanical:  25 traditional medicine healers were interviewed.  *In vitro*:  *S. aureus* (ATCC25923), *E. feacalis* (ATCC29212), E. coli (ATCC25922) and *S. typhimurium* (ATCC13311) | *Allium cepa* L*.*  *Allium sativum* L.  *Foeniculum vulgare* Mill.  *Lepidium sativum* L.  *Artemisia afra* Jacq. ex Willd.  *Vernonia amygdalina* Delile  *Croton macrostachyus* Hochst. ex. Delile  *Ricinus communis* L.  *Hydnora Abyssinia* A. Br  *Leucas aspera* (Willd.) Link.  *Ocimum lamiifolium* Hochst. ex Benth  *Linum usitatissimum* L.  *Moringa stenopetala* (Baker f.) Cufod  *Syzygium guineense* (Willd.) DC.  *Phytolacca dodecandra* L, H, er.  *Rumex abyssinicus* Jack  *Nigella sativa* L.  *Hagenia abyssinia* (Bruce ex Steud.) J.F. Gmel  *Citrus aurantifolia* (Christm.) Swingle  *Citrus sinensis* (L.) Osbeck  *Harrisonia abyssinica* Oliv.  *Ruta chalepensis* L.  *Capsicum annuum* L.  *Solanum incanum L*  Withania somnifera (L.) Dunal  *Zingiber officinale* Roscoe | About 100 g of the powdered extract of each plant sample was extracted by maceration in five solvent systems (aqueous, chloroform, ethanol, acetone and ethyl acetate) within 72 h and filtered | Gentamicin | *H. abyssinica Oliv., S. incanum L., H. abyssinia,* and *L. aspera* were found to be the most frequently used medicinal plants in the study area. These plants showed a promising broad spectrum of activity against Gram-positive and Gram-negative test bacteria with growth inhibition zone and minimum inhibition concentration (MIC) values ranging from 7.40 ± 0.60 − 16.81 ± 2.03 mm and 156 − 2500 μg/ML, respectively |
| 1. Combination (Ethnobotanical and *in vitro*) | [61] | Not reported | Kenya | Ethnobotanical:  A survey was carried out in Wamba division, Samburu district.  *In vitro*:  *S. aureus* ATCC 20591, *B. subtillis, S. typhi* ATCC 2202, *E. coli* STD-25922 and *P. aeruginosa* ATCC 25852 | *Acacia ethaaica* Schweinf.  *Acacia horrida (L.)* Willd.  *Acacia nilotica* (L.) Del.  *Acacia nubica* Benth.  *Acacia senegal* (L.) Willd.Var. persica  *Acacia tortilis* (Forssk.) Hayne.  *Acokanthera friesiorum* Markgr.  *Albizia anthelmitica* Brongn.  *Aloe secundiflora* Engl.  *Balanites aegyptiaca* (L.) Del.  *Boscia angustifolia* Guill. and Perr.  *Cissus rotundifolia* Forsk. Vahl.  *Cissus quadrangularis L.*  *Clerodendrum myriacoides (Hochst.)* Vatke subsp. napperae Verdc  *Commiphora africana* (A. Rich) Engl. Var. persica  *Cordia monoica* Roxb. | Dried, ground plant materials (50g) were soaked in 300 ml of 80% methanol for 12-48h and filtered | Amoxicillin | Phytochemical screening revealed that the extracts had a good number of the most active phytochemicals such as tannins, saponins, flavonoids, and terpenoids. In general, most of the extracts produced good MICs and MBCs. *A. ethaaica* Schweinf., *A. nilotica* (L.) Del., *A. horrida* (L.), and *A. tortilis* showed great activity producing MICs and MBCs ranging from 0.9375 mg/50µl to 1.875 mg/50µl in the test cultures |
| 1. Combination (Ethnobotanical and *in vitro*) | [130] | Public | South Africa | Ethnobotanical:  Traditional healers, herbalists, elderly people and the young men and women were interviewed  *In vitro*:  *E. coli* (ATCC 11775), *S. flexneri* (ATCC 12022) *and S. aureus* (ATCC 12600) | *Aloe ferox* Mill.  *Brunsvigia grandiflora* Lindl.  *Searsia chirindensis* (Baker f.) Moffett  *Aster bakerianus* Burtt Davy ex C.A.Sm.  *Berkheya bipinnatifida* (Harv.) Roessler subsp. Bipinnatifida  *Senecio serratuloides* DC.  *Tecoma capensis* (Thunb.) Lindl.  *Cyperus dives* Delile  *Clutia pulchella* Spam. ex Sond*.*  *Alysicarpus* rugosus (Willd) DC.  *Pelargonium luridum* Sweet  *Eucomis autumnalis* (Mill.) Chitt.  *Ledebouria ovatifolia* (Baker) Jessop  *Hydnora africana* Thunb.  *Hypoxis hemerocallidea* Fisch. C.A. Mey. and Avè-Lall  *Ocotea bullata* (Burch.) Baill.  *Malva parviflora* L.  *Turraea obtusifolia* Hochst.  *Acacia mearnsii* De Wild.  *Elephantorrhiza elephantina* (Burch.) Skeels  *Ficus craterostoma* Warb. ex Mildbr. and Burret  *Maesa lanceolata* Forssk.  *Rapanea melanophloeos* Mez  *Eucalyptus camaldulensis* Dehnh.  *Psidium guajava* L.  *Ziziphus mucronata* Willd*.*  *Prunus persica* (L.) Stokes  *Pentanisia prunelloides* (Klotzsch) Walp  *Zanthoxylum capense* (Thunb.) Harv.  *Physalis peruviana* L.  *Solanum aculeastrum* Dunal  *Dais cotinifolia* L.  *Trema orientalis* (L.) Blume  *Rhoicissus tridentata* (L.f.) Wild and R.B. Drumm | The ground plant material (10 g) was extracted non-sequentially with 200 ml of DCM, PE, 70% EtOH and water by sonication for 1 h. The extracts were then filtered | Neomycin (100 μL) | This study revealed that 34 plant species belonging to 27 families are used for the treatment of diarrhoea in Bizana. The extracts showed good inhibitory activity with MIC values ranging from 0.39 to 12.5 mg/ml. The best activity was exhibited by DCM extracts of *R. melanophloeos*, and EtOH extracts of *F. craterostoma* and *M. lanceolata* with MIC values of 0.098 mg/ml |
| 1. *In vitro* | [131] | Not reported | South africa | The bacterial species used*, P. vulgaris* CSIR 0030*, P. vulgaris* *CSIR, Shigella flexneri, Micrococcus luteus, Enterococcus faecalis KZN, Staphylococcus aureus* OK_3_*, Shigella sonnei, Escherichia coli, Bacillus pumilus, Enterococcus faecalis, Pseudomonas aeruginosa, and Staphylococcus aureus* | *Erythrina caffra* Thunb. | Stem bark air dried, powdered, extracted with ethanol and filtered | Ciprofloxacin | The crude ethanolic extract of *E. caffra* demonstrated significant inhibitory and bactericidal effects against all the test bacteria isolates. *Micrococcus luteus, Proteus vulgaris* CSIR 0030, and *Staphylococcus aureus* OK_3_ were the most highly affected bacteria |
| 1. *In vitro* | [125] | Not reported | Uganda | Third-generation cephalosporin-resistant *Escherichia coli, methicillin-resistant Staphylococcus aureus, multidrug-resistant P. aeruginosa, ciprofloxacin-resistant Salmonella typhimurium,* and methicillin-sensitive *Staphylococcus aureus* ATCC®25923™ | *Artocarpus heterophyllus* | Seeds macerated, powdered, extracted in ethanol and absolute hexane, and filtered | Ceftriaxone (15 μg) and vancomycin (30 μg) | Ethanolic and hexanolic extracts had activity on multidrug-resistant P. aeruginosa, methicillin-resistant *Staphylococcus aureus*, and methicillin-susceptible *S. aureus* with the mean and standard error zone of inhibition that ranged from 8.5 ± 0.5 to 16.5 ± 0.25 mm; however, the extracts were found not to have activity on resistant *E. coli* and *S. typhimurium* |
| 1. *In vitro* | [126] | Public | South Africa | The most important nosocomial pathogens including two Gram positive species (*Staphylococcus aureus* (ATCC 29213), *Enterococcus faecalis* (ATCC 29212)), and two Gram negative (*Escherichia coli* (ATCC 25922), *Pseudomonas aeruginosa* (ATCC 27853) | *Maytenus peduncularis*, Maytenus *procumbens*, Maytenus *senegalensis* (syn *Gymnosporia senegalensis* (Lam.) Loe) and *Maytenus undata* | Ground leaf materials extracted, crude extracts and fractions with varying polarities using various solvents | Gentamicin | The crude extracts and polar fractions (butanol and water) had moderate to poor antimicrobial activity (MICs 312 to above 2500 μg/ml). Many of the extracts and fractions were not highly active against microbes tested in this work |
| 1. *In vitro* | [72] | Public | South Africa | Three Gram-positive and three Gram-negative bacteria (*Staphylococcus aureus, Enterococcus faecalis, Bacillus cereus, Pseudomonas aeruginosa, Salmonella Typhimurium*, and *Escherichia coli*) | *Hypericum roeperianum, Cremaspora triflora, Heteromorpha arborescens, Pittosporum viridiflorum, Bolusanthus speciosus, Calpurnia aurea, Maesa lanceolata, Elaeodendron croceum,* and *Morus mesozygia* | Leaf powder extracted with acetone | Gentamicin | The extracts were active against all the pathogens with average MICs ranging from 0.02 to 0.52 mg/ml. Extracts of *M. lanceolata and H. roeperianum* had the highest total antibacterial activity at 1417 and 963 ml/g respectively |
| 1. *In vitro* | [132] | Public | South Africa | Surface water borne isolate *Klebsiella pneumoniae, commercial probiotics (combination of Lactobacillus acidophilus, Lactobacillus casei, Lactobacillus plantarum, Lactobacillus rhamnosus, Lactobacillus salivarius, Lactobacillus bifidum, Lactobacillus breve, Lactobacillus lactis, and Lactobacillus thermophillus), E. coli* ATCC25922™, *Salmonella typhimurium* ATCC13311™, *Shigella boydii* ATCC9207™ and *Vibrio parahaemolyticus* ATCC17802™ | *Bidens pilosa* Linn. *Dichrostachys cinerea* Wight et Arn | Two and five grams of powdered leaves were separately mixed with 20 ml and 50 ml of hexane, dichloromethane, ethyl acetate, acetone and methanol according to the solvents polarities from non-polar to the more polar solvent, and filtred | Ampicillin (1 mg/ml) | Phytochemical profiling of the extracts displayed the presence of various secondary metabolites. All the bacterial species tested were sensitive to the effect of different extracts of both plant species, with *E. coli* being less sensitive to the effect of the extracts from *D. cinerea* |
| 1. *In vitro* | [127] | Not reported | Kenya | *Bacillus subtilis* BGA, *Bacillus cereus* (ATCC 11778),  *Staphylococcus aureus* ATCC 25923 (KEMRI), (*Escherichia coli* ATCC 25922 (KEMRI), *Pseudomonas aeruginosa* ATCC 27853 (KEMRI), *Salmonella typhimurium* ATCC 13311 (KEMRI), *Klebsiella pneumoniae* and *Proteus mirabilis* (clinical isolates from KEMRI) | *Indigofera lupatana* Baker F. | Powdered sample of roots were extracted using methanol solvent | Chloramphenicol | The extract showed the highest activity against *B. subtilis* (28.0 mm) and *B. cereus* (22.0 mm). A variety of phytochemicals such as tannins, saponins, terpenoids, cardiac glycosides, phytosteroids, phlobatannins, and flavonoids were detected in the plant methanol extracts |
| 1. *In vitro* | [56] | Not reported | Sudan | *E. coli* ATCC 25922, *S. aureus* ATCC 25923 and ATCC 29213, *E. faecalis* ATCC 29212, *P. aeruginosa* ATCC 27853, *B. subtilis* ATCC 6633 and *K. oxytoca* ATCC 700324, *Corynebacterium jeikeium* S1, *Listeria monocytogenes* S1, *E. coli* O55 S1, *Proteus vulgaris* S1, *Salmonella enterica Anatum* S1, S. *enterica Enteritidis* S1, *S. enterica Enteritidis* S2, S. *enterica Newport*, S. *enterica Typhimurium* S1, *S. enterica Typhimurium* S2, *S. enterica Typhimurium* S3, *Shigella boydii* S1, *S. flexneri* S1, S. *sonnei* S1, *Yersinia enterocolitica* S1, *Y. enterocolitica* S2 | *Hydnora johannis* | Two main types of extracts (water and ethanol) were prepared and then were further subjected to different fractionations. Water extract (WE) was prepared by simple maceration and ethanolic extract was prepared by soaking | Amoxicillin, penicillin G, ticarcillin, and vancomycin | The water extract was found to possess antibacterial activity against *E. faecalis* (MIC value of 16 μg/mL), *B. subtilis, B. cereus and S. aureus* (MIC values of 64 μg/ml) but not against bacteria mainly responsible for diarrhea |
| 1. *In vitro* | [65] | Public | South Africa | *S. typhi, S. enterica serovar Typhimurium, Shigella flexneri* type 1b and Sh. sonnei phase II and typed culture of *Escherichia coli* (ATCC 25922), *E. faecalis* (ATCC 29212), *S. aureus* (ATCC 29213) | *Acacia mearnsii* De Wild., *Aloe arborescens* Mill., Aloe striata Haw., *Cyathula uncinulata* (Schrad.) Schinz, *Eucomis autumnalis* (Mill.) Chitt, *Eucomas* comosa (Houtt.) Wehrh., *Hermbstaedtia odorata* (Burch. ex Moq.) T. Cooke, *Hydnora africana* Thunb., *Hypoxis latifolia* Wight*, Pelargonium sidoides* DC., *Psidium guajava* L., *Schizocarphus nervosus* (Burch.) van der Merwe. | Fine powder soaked overnight in 500 ml of acetone on an orbital shaker, and filtered | Gentamycin | The TLC fingerprint indicated the presence of terpenoids and flavonoids in the herbs. Most of the tested organisms were sensitive to the crude acetone extracts with MIC values ranging from 0.018–2.5 mg/ml |
| 1. *In vitro* | [96] | Not reported | Malawi | *Escherichia coli, Pseudomonas aeruginosa, Staphylococcus aureus* and *Candida albicans* | *Dalbergiella nyasae* | Finely powdered sample was extracted three times with 10 ml of solvents (acetone, ethanol and distilled water), maceration, and fractionated using solvents of varying polarities | Chloromphenical and fluconazole | Phytochemical analysis demonstrated the presence of alkaloids, flavonoids, saponins and terpenoids. Excellent antimicrobial activity was demonstrated by n-butanol fraction of leaves extract by showing low MIC and MBC/MFC values for *P. aeruginosa* and *C. albicans* |
| 1. *In vitro* | [87] | Public | South Africa | *B. cereus* (ATCC 11778), *E. faecalis* (ATCC 29212), *E. coli* (ATCC 8739), *P. vulgaris* (ATCC 33420), *S. typhimurium* (ATCC 14028), *S. flexneri* (ATCC 25875) and *S. aureus* (ATCC 12600) | *Acacia burke* Benth,  *Acanthospermum glabratum* (DC) Wild, *Brachylaena Transvaalensis* E. Phillips & Schweick, *Catharanthus roseus* (L.) G. Don, *Chenopodium ambrosioides* L., *Cissampelos hirta* Klotzch, *Garcinia livingstonei* T. Anderson, *Gymnosporia senegalensis* (Lam.) Loes., *Krauseola mosambicina* (Moss.) Pax & K. Hoffm., *Lippia javanica* (Burm.f.) Spreng., *Mangifera indica* L., *Melia azedarach* L., *Psidium guajava* L., *Sarcostemma viminale* (L) R. Br subsp. viminale, *Schotia brachypetala* Sond. *Sclerocarya birrea* (A. Rich.) Hochst. subsp. caffra (Sond.), *Senna occidentalis* (L.) Link, *Strychnos madagascariensis* pior., *Syzygium cordatum* Hochst. ex. C. Krauss., *Terminalia sericea* Burch. ex DC., *Trichilia emetica* Vahl, *Vangueria infausta* Burch. subsp. infausta, *Vernonia natalensis* (DC) Sch. Bip. ex. Walp | Two types of plants extractions were prepared, a 1:1 mix of dichloromethane: methanol (organic) and an aqueous extract for each plant species; heated and filtered | Ciprofloxacin (0.02 mg/ml) | *S. flexneri* proved to be the most susceptible pathogen, where the organic extract of *T. sericea* showed the most prominent noteworthy antibacterial activity (mean MIC value of 0.04 mg/l). The aqueous extracts generally showed poorer antimicrobial activity with some exceptions i.e. *A. burkei, B. transvaalensis against B. cereus* and *B. transvaalensis* against *S. flexneri* |
| 1. *In vitro* | [97] | Not reported | Tanzania | *S. aureus* (NCTC 6571), *E. coli* (NCTC 10418), *P. aeruginosa* (NCTC 10662), *S. typhi* (NCTC 8385), *B. cereus* (NCTC 7464), *P. mirabilis* (NCTC 10975), *S. flexineri* (clinical isolate), *V. cholerae* (clinical isolate) and two fungi, *C. albicans* (Strain HG 392), *C. neoformans* (clinical isolate) | *Canarium schweinfurthii* Engl., *Dissotis brazzae* Cong, *Iboza urticifolia* (Bak) E.A. Bruce, *Isoglosa lacteal* Lindau, *Strombosia scheffleri* Engl., *Whitfieldia elongate* T. Anders | Powders soaked for 48h, with dichloromethane, ethyl acetate and ethanol. The extracts were dried and freeze dried | Cyclophosphamide (for toxicity assessment) | At least one extract of each plant showed antibacterial activity. DCM extracts were the most active while ethanol extracts were the least active. Extracts of *W. elongate* and *I. lacteal* were the most and least active with MICs in the range 0.08-0.62 mg/ml and 15.6-62.5 mg/ml, respectively |
| 1. *In vivo* | [73] | No funding | Ethiopia | Swiss albino mice of either sex weighing 20 to 30 g and aged 6 to 8 weeks. Mice were randomly assigned into 5 groups of 3 extracts treated and 2 control groups with 5 mice per group | *Dodonaea viscosa* L. | A total of 200 g of coarsely powdered leaves were macerated with 80% methanol in Erlenmeyer flask for 72 hours | Loperamide (3 mg/kg) | At all test doses, the plant extract showed significant (P < .05) inhibition in the frequency of defecation of wet feces and total fecal output as compared to the control group |
| 1. *In vivo* | [128] | Public | Ethiopia | Swiss Albino mice of both sexes weighing (31.25 + 0.74 g). The animals were randomly assigned to 5 groups, each consisting of 6 mice | *Osyris quadripartita* Decne | Powder, 600 g plant extract material was macerated in 2400 ml of 80% methanol for 72 hours | Loperamide | The extract at all tested doses resulted in significant reduction (P < .01) in number of wet feces, whereas significant reduction (P < .01) in frequency of defecation in castor oil–induced diarrhea was seen at a dose of 400 mg/kg |
| 1. *In vivo* | [133] | Not reported | South Africa | Male and female *Balb* C mice (*Mus domesticus*) weighing 20–25 g, and healthy young adult, Wistar rats (*Rattus norvegicus*) of both sexes weighing 250–300 g. The animals were divided into three groups of 8-10 animals per group | *Psidium guajava* Linn. | The powdered leaf was macerated in distilled water and extracted twice, on each occasion with 2.5 L of distilled water for 48 hours | Loperamide (10 mg/kg) | Like loperamide (10 mg/kg), PGE dosedependently and significantly (P<0.05-0.01) delayed the onset of castor oil-induced diarrhoea, decreased the frequency of defaecation, and reduced the severity of diarrhoea in the rodents |
| 1. *In vivo* | [134] | Not reported | Nigeria | New Zealand rabbits weighing 1.5 kg and male Swiss albino mice weighing 22.0 ± 0.5 g. The mice were randomly divided into four groups of five mice each | *Acacia nilotica, Acanthospermun hispidum, Gmelina arborea, Parkia biglobosa* and *Vitex doniana* | The powdered parts of selected plants were macerated in aqueous methanol | Loperamide (5 mg/kg) | In the castor oil-induced diarrhoeal, 100% protections were shown by extracts of *A. nilotica* and *P. biglobosa* (100, 200 mg/kg) while *V. doniana* showed a dose-dependent effect. The least protection was shown by *A. hispidum*, at the same dose, when compared with the other four plants |
| 1. *In vivo* | [74] | Public | Benin | Three-month-old Wistar albino rats weighing between 130 and 180 g, all nonpregnant and female. The rats were randomly assigned to groups of 3 | *Khaya senegalensis, Daniellia oliveri, Rauvolfia vomitoria, Vernonia amygdalina, Manihot esculenta, Ocimum gratissimum, Senna italica, Diospyros mespiliformis, Pterocarpus erinaceus,* and *Anacardium occidentale* | Fifty (50) grams of powder were macerated in 500 ml of solvent (water and water-ethanol). The mixture was stirred continuously for 72 hours | Distilled water | The qualitative phytochemical screening of the plants studied revealed the presence of catechic tannins, gallic tannins, flavonoids, anthocyanins and sterol-terpenes, alkaloids, saponosides, and reducing compounds |
| 1. *In vivo* | [135] | Not reported | Nigeria | Healthy male albino Wistar rats (100–150 g). The animals were fasted for 18 h and divided into 5 groups of 5 animals each | *Citrus limon* | The coarse powder of the peels was soaked in hexane in a 500 ml flat bottom reagent bottle. It allowed to stand for 10 d with occasional shaking and stirring. | Loperamide (3 mg/kg) | Each of the 3 doses of C. limon significantly reduced (P < 0.05) the number of wet fecal pellets produced by animals, with 20 mg/kg of the plant extract producing the highest percentage inhibition (34.2%). |
| 1. *In vivo* | [75] | Public | Uganda | Wistar albino rats, both male and female, weighing between 150 and 200 g were randomly divided into 5 groups of 6 rats each | *Priva adhaerens* | Fine powder (100 g) of air-dried leaves of *P. adhaerens* was subjected to the Soxhlet extractor for continuous hot extraction with distilled water. As quality control, the aqueous extract was also obtained by maceration through soaking the powder overnight in distilled water, then freeze-drying the extract | Loperamide (5 mg/kg) | Aqueous extract significantly, and dose-dependently, reduced frequency of stooling in castor oil-induced diarrhea, intestinal motility, and castor oil-induced enteropooling in rats |
| 1. Cross- sectional | [12] | Public | Uganda | Was conducted on 65 herbalists | *Thunbergia alata* Bojer ex Sims  *Pseudospondias microcarpa* (A. Rich) Engl.  *Annona senegalensis* Pers  *Mondia whytei* (Hook.f.) Skeels  *Aristolochia littoralis* Parodi  *Microglossa angolensis* Oliv. & Hiern  *Conyza pyrrhopappa* Sch. Bip. ex A. Rich  *Microglossa pyrifolia* (Lam.) O. Kuntze  *Solanecio mannii (Hook.f.)* C. Jefrey  *Bidens pilosa* L.  *Balanites aegyptiaca* (L) Delile  *Canarium schweinfurtii* Engl.  *Capparis tomentosa* Lam.  *Ipomoea batatas* (L.) Lam  *Bryophyllum pinnatum* (Lam.) Oken  *Euphorbia heterochroma* Pax  *Abrus precatorius* L.  *Acacia amythethophylla* A. Rich.  *Acacia senegal* (L.) Willd. S  *Albizia coriaria* Oliv  *Cajanus cajan* (L.) Millsp.  *Erythrina abyssinica* DC.  *Tylosema fassoglensis* (Schweinf.) Torre & Hillc.  *Punica granatum* L.  *Lovoa trichilioides* Harms  *Ficus natalensis* Hochst.  *Eucalyptus globulus* Labill.  *Eucalyptus saligna* Sm.  *Oxalis latifolia* Kunth  *Clutia abyssinica* Jaub. & Spach  *Bridelia micrantha* (Hochst.) Baill  *Phyllanthus ovalifolus* Forssk.  *Cymbopogon fexuosus* (Nees ex Steud.) W. Watson  *Digitaria abyssinica* (A. Rich.) Stapf  *Securida longipedunculata* Fresen.  *Maesa lanceolata* Forssk.  *Prunus africana* (Hook.f.) Kalkman  *Eriobotrya japonica* (Thumb) Lindl.  *Ocimum gratissimum* L.  *Citropsis articulata* (Willd. ex Spreng.) Swingle & M. Kellerm  *Priva fabelliformis* (Moldenke) R. Fern.  *Zingiber ofcinale* Roscoe  *Mangifera indica* L.  *Searsia pyroides* (Burch.) Mofett  *Warburgia ugandensis* Sprague  *Momordica foetida* Schumach  *Azadirachta indica* A. Juss  *Passifora edulis* Sims  *Cymbopogon citratus* Stapf.  *Citrus sinensis* (L.) Osbeck  *Entada abyssinica* A. Rich | Decoction  Decoction  Decoction  Infusion/chew  Infusion/chew  Decoction  Decoction  Decoction  Decoction  Decoction  Decoction  Decoction  Decoction  Decoction  Decoction  Decoction  Decoction  Decoction  Decoction  Decoction  Decoction  Decoction/infusion  Decoction  Decoction  Concoction  Infusion  Decoction  Decoction  Decoction  Decoction  Decoction  Infusion/decoction  Decoction  Decoction  Infusion  Decoction  Concoction  Decoction  Decoction  Decoction  Decoction  Decoction  Tincture  Decoction  Decoction  Decoction/infusion  Infusion  Decoction  Decoction  Decoction/infusion  Decoction  Decoction/infusion | N/A | N/A |
| 1. Cross- sectional | [109] | Not reported | Tanzania | A total of 161 mothers were interviewed. Of those, 74 (46%) had female and 87 (54%) had male underfives with median age of 2‐years. | *Psidium guayava*  *Mangifera indica, Annona senegalensis, Anacardium accidentale, Punica granatum, Solanum incanum, Cajanus cajani* and *Tamarindus indica* | Leaves or fruits were boiled, and then upon cooling, the liquid was administered orally to children | N/A | N/A |
| 1. Cross- sectional | [104] | Not reported | Uganda | A total of 202 participants were interviewed (healers, herbal medicine gatherers and users of medicinal plants). Females (57.9%) and males (42.1%) | *Chenopodium ambrosioides L.*  *Rhus vulgaris Meikle*  *Carissa edulis Vahl.*  *Ageratum conyzoides L.*  *Bothriocline longipes (Oliv. & Hiern) N.E.Br.*  *Guizotia scabra* Chiov.  *Vernonia amygdalina* Delile  *Diospyros abyssinica* (Hiern) F. White.  *Jatropha curcas* L.  *Pseudarthria hookeri* Wight & Arn.  *Leonotis nepetifolia* (L.) R.Br.  *Plectranthus barbatus* Andrews  *Persea americana* Mill.  *Azadirachta indica* A. Juss.  *Dichrostachys cinerea* (L.) Wight & Arn.  *Psidium guajava* L.  *Digitaria abyssinica* (Hochst.) Stapf.  *Sorghum bicolor* (L.) Moench  *Dodonaea angustifolia* L.f.  *Harrisonia abyssinica* Oliv.  *Lantana trifolia L.* | Boiling  Pound and add water (twice daily)  Pound and add water  Pound and add water  Pound and add water (adult)/1 spoonful (children) thrice daily  Pound, add water (boil)  Squeeze/pound, add water or boil and drink; ½ glass (child), 1 glass (adult) thrice daily  Boiling  Pound, add water or boil  Pound, add water and sieve  Pound (with charcoal), add water and take 1 glass twice daily  Pound, add water OR boil (with cow ghee) and drink thrice daily  Boiling (thrice daily)  Boil and drink 500 ml 3xdaily  Pound 2 handfuls, boil and sieve while still warm and drink  300 ml (adult), 150 ml (child)  Pound, add water or boil (with rock salt) and drink 250 ml  Pound, add water or boil and drink 1 glass thrice daily  Pound, add water and drink 500 ml  Chew and swallow  Pound, add water or boil and drink 500 ml daily  Pound, add water or boil and drink 1 glass daily | N/A | N/A |

Minimum inhibitory concentration: MIC, Minimum Bactericidal Concentration: MBC, minimum fungicidal: MFC, dichloromethane: DCM, petroleum ether: PE, ethanol: EtOH, *Bacillus cereus: B. cereus, Enterococcus faecalis: E. faecalis, Escherichia coli: E. coli, Proteus vulgaris: P. vulgaris, Salmonella typhimurium: S. typhimurium, Shigella flexneri: S. flexneri, Staphylococcus aureus: S. aureus, Pseudomonas aeruginosa: P. aeruginosa, Proteus mirabilis: P. mirabilis, Vibrio cholera: V. cholera, Candida albicans: C. albicans, Cryptococcus neoformans: C. neoformans, lmonella typhy: I. typhy,*

| **Author**  **Supplementary Table S3**: Summary of included ethnobotanical information studies using medicinal plants as antidiarrhoeal treatment. | **Country** |  | **Subject** | **Intervention details** | | |
| --- | --- | --- | --- | --- | --- | --- |
| **(Year)** |  | **Funding** |  | **Species name** | **Preparation** | **Application**  **route** |
| [136] | Nigeria | Not reported | Thirty homes in each of the local government area were visited resulting in the interview of 930 households (one interviewee per household). | *Achyranthes aspera L.*  *Uvaria chamae P. Beauv.*  *Croton zambesicus Mull. Arg.*  *Gnetum africanum Welw.* | Infusion  Decoction or macerated in soda water as infusion  Boiled in water to make decoction  Chewed or cooked as soup | Oral  Oral  Oral  Oral |
| [116] | Ethiopia | Not reported | Mainly through individual interviews with the selected informants using semi-structured interview format | *Ajuga integrifolia Buch. Ham. ex D. Don*  *Clerodendrum myricoides (Hochst.) Vatke*  *Cyperus sp.*  *Hoslundia opposita Vah*  *Leucas deflexa Hook.f.*  *Pentas lanceolata (Forssk.) Deflers*  *Rubus steudneri Schweinf* | NR | Oral  Nasal  Oral  Oral  Nasal  Oral  Oral |
| [137] | South Africa | Not reported | Seventy-four community members including women, traditional healers and farmers between the ages of 20 and 80 years participated in the study. This consisted of 35 males (47%) and 39 females (53%) | *Amaranthus hybridus L*  *Syzygium cordatum (Hochst.) ex Krauss* | Decoction  Decoction | NR  NR |
| [138] | Lesotho | Not reported | Local practising herbalists who were willing to participate in the study. | *Dicoma anomala Sond.*  *Eucalyptus sideroxylon* A. Cunn.  *Kedrostis capensis* (Sond.) A. Meeuse  *Ledebouria marginata* (Baker) Jessop.  *Pelargonium sidoides* (L.) L'Hér.  *Rhus erosa Drege* ex C. Presl  *Senecio harveianus L. Xysmalobium undulatum R.Br.* | Add powder to water  Breathe fumes  Decoction  Decoction  Crush & boil  Decoction  Crush & add water  Boil powder | NR  NR  NR  NR  NR  NR  NR  NR |
| [58] | Congo | Not reported | Traditional healers and people of Lomela area, mainly in the villages where epidemics of diarrhoea and dysentery occurred | *Epinetrum Villosum.* (Excell) Troupin  *Roureopsis obliquifoliolata.* (Gild) Schellenb  *Croton mubango. Müll.*  *Cissus rubiginosa* (Welw Ex. Bak) *Planch*  *Vernonia amygdalina.* Del  *Quassia africana.* Baill | Decoction  Decoction  Maceration  Decoction  Maceration  Decoction | Oral  Oral  Oral  Oral  Oral  Oral |
| [139] | South Africa | Public | Traditional healers | *Acacia nigrescens* | Infusion | Oral |
| [140] | South Africa | Public | Several local experts | *Artemisia absinthium L.*  *Atriplex nummularia Lindl.* | Leaves as tea  Drink a leaf infusion | NR |
| [141] | Sudan | Not reported | Two hundred and fifty-eight informants including 16 herbalists, 195 male and 63 were interviewed | *Sclerocarya birrea* (A. Rich.) Hochst.  *Adansonia digitata L.*  *Guiera senegalensis* J.F. Gmel. | Maceration  Maceration and fresh leaves (mixed with sesame seeds)  Decoction | NR  NR  NR |
| [118] | Mozambique | Not reported | Eleven informants (six men and five women) were selected as the best traditional knowledge holders | *Lannea schweinfurthii (Engl.) Engl.*  *Sclerocarya birrea (A. Rich.) Hochst*  *Adansonia digitata L.*  *Boscia albitrunca (Burch.)* Gilg & Gilg-Ben.  *Maerua parvifolia* Pax  *Terminalia sericea Burch.* ex DC.  Cassia abbreviata Oliv.  *Colophospermum mopane* (Benth.) Léonard | Decoction  Scraping and decoction  Maceration  Crushing and infusion  Decoction  Decoction  Decoction  Infusion | Oral  Oral  Oral  Topic  Oral  Oral  Oral  Oral |
| [142] | Ethiopia | Public | A total of 75 informants were involved in the study | *Acacia albida* Del*.*  *Calpurnia aurea* (Ait.) Benth  *Carica papaya* L.  *Melia azedarach* L.  *Punica granatum* L.  *Rumex nervosus* Vahl.  *Sena obtusifolia* (L.) Irwin and Barneby  *Verbascum sinaiticum* Benth | Crushed  Crushed  Crushed and swallowed  Crushed  Boiled and drunk  Crushed  Crushed  Crushed | Oral  Oral  Oral  Oral  Oral  Oral  Oral  Oral |
| [143] | South Africa | Public | 30 traditional healers (24 females and 6 males) in the Lwamondo area of Venda | *Diospyros mespiliformis* | Boiled and the blend | Oral |
| [59] | Zimbabwe | Not reported | Nine traditional healers were interviewed | *Albizia antunesiana* Harms  *Ampelocissus africana* (Lour.) Merr.  *Ampelocissus obtusata* (Welw. ex Baker) Planch.  *Asparagus africanus* Lam.  *Carissa bispinosa* (L.) Desf. ex Brenan  *Carisa edulis* (Forssk.) Vahl  *Cassia abbreviata* Oliv.  *Elephantorrhiza goetzei* (Harms) Harms  *Euclea divinorum* Hiern  *Ficus sur* Forssk.  *Flacourtia indica (Burm. f.) Merr.*  *Grewia bicolor Juss.*  *Grewia monticola Sond.*  *Lannea edulis (Sond.) Engl.*  *Moringa oleifera Lour*  *Ozoroa insignis Del.*  *Peltophorum africanum Sond.*  *Vangueria infausta Burch.*  *Ximenia caffra Sond.* | Crushed and mixed with hot water  Crushed and mixed with water  Crushed and mixed with hot water  Crushed and mixed with hot water  Hot water extract drunk three times a day  Crushed and mixed with hot water  Crushed and mixed with hot water  Hot water extract drunk three times a day  Hot water extract drunk three times a day  Crushed and mixed with hot water  Leaves browsed by mouth  Hot water extract drunk three times a day  Hot water extract drunk three times a day  Hot water extract drunk three times a day  Crushed and mixed with hot water  Hot water extract drunk three times a day  Hot water extract drunk three times a day  Hot water extract drunk three times a day  Crushed and mixed with hot water | Oral  Oral  Oral  Oral  Oral  Oral  Oral  Oral  Oral  Oral  Oral  Oral  Oral  Oral  Oral  Oral  Oral  Oral  Oral |
| [121] | Mozambique | Public | A total of 25 informants were interviewed (14 women and 11 men), their ages ranging from 40 to 86 years old (average: 57,3 ± 13,5). | *Hymenocardia acida* Tul.  *Margaretta rosea* Oliv.  *Ozoroa obovata* (Oliv.) R. Fern. & A. Fern.  *Senna petersiana* (Bolle) Lock | The bark is cut, the exudate is collected and mixed with water to give to the baby  Dig roots, cut into small pieces, put in water and let them rest for two hours, then drink the water.  Maceration  Dig roots, cut into small pieces, put in water and let them rest for one hour, drink the water (Very effective). | Oral  Oral  Oral  Oral |
| [165] | Ethopia | Public | 17 knowledgeable elders (16 men and 1 woman) between the ages of 41 and 77 (3 from Tullu Guddo, 4 from Mekdela and 10 from Bochesa), chosen with the assistance of local administrators and community leaders, served as key informants | *Eleusine coracana (L.) Gaertn.* | NR | Oral |
| [144] | Congo | Not reported | Traditional healers, young and old people of both sexes were interviewed | *Crassocephalum montuosum.* (S. Moore) Milne-Redh.  *Helichrysum forskhalii* (J.M. Gmel.) Hilliard A  *Indigofera colutea* (Burm. F)  *Momordica foetida Schumch.* et Thonn.  *Musa acuminata* Colla  *Ocimum basilicum* L.  *Ocimum lamiifolium Hochst.* ex Benth.  *Secamone stuhlmannii* K. Schum.  *Zanthoxylum gilletii* (De Wild.) P.G. | Maceration (once a day)  Maceration (taken once a day)  Maceration (twice a week for 1 week)  Maceration (twice a day for 1 week)  Maceration (thrice a day)  Crushed and mixed with water to make a maceration (thrice a day)  Decoction (twice a day)  Maceration  Maceration | Oral or rectal  Oral  Rectal  Rectal  Oral  Oral  Oral  Rectal  Rectal |
| [145] | Madagascar | Public | 193 informants from 16 to 86 years old were interviewed, of which 54 % were men and 46 % were women. Most of them are farmers. | *Litchi chinensis* Sonn*.*  *Rubus moluccanus* L*.*  *Psidium guajava* L.  *Clidemia hirta* (L.) D. Don  *Harungana madagascariensis* Lam. ex Poir.  *Sorindeia madagascariensis* DC.  *Cocos nucifera* L.  *Raphia farinifera* (Gaertn.) Hyl.  *Elephantopus scaber* L.  *Psiadia altissima* (DC.) Drake  *Canarium L.*  *Symphonia fasciculata (*Noronha ex Thouars) Vesque  *Cnestis polyphylla* Lam.  *Macaranga obovata Boivin* ex Baill*.*  *Manihot esculenta* Crantz  *Desmodium ramosissimum* G. Don  *Entada gigas* (L.) Fawc. & Rendle  *Exacum quinquenervium* Griseb.  *Sticherus flagellaris* (Bory ex Willd.) Ching  *Harungana madagascariensis* Lam. ex Poir  *Plectranthus perrieri* Hedge  *Clidemia hirta* (L.) D. Don  *Artocarpus heterophyllus* Lam.  *Streblus dimepate (Bureau)* C.C. Berg  *Musa paradisiaca* L.  *Eucalyptus camaldulensis* Dehnh.  *Psidium cattleyanum* Sabine  *Psidium guajava L.*  *Syzygium malaccense* (L.) Merr. & L.M. Perry  *Passiflora edulis* Sims  *Maesa lanceolata* Forssk.  *Rubus moluccanus L.*  *Danais terminalis Boivin* ex Drake  *Litchi chinensis* Sonn.  *Lycopersicon esculentum* Mill.  *Solanum mauritianum* Scop.  *Lantana camara* L. | Decoction  Crush and decoction  Decoction  Decoction  Decoction  Decoction  Infusion  Decoction  Crush and heat  Decoction  Decoction  Decoction  Decoction  Decoction  Decoction  Decoction  Decoction  Decoction  Decoction  Decoction  Decoction  Decoction  Crush  Crush  Paste  Decoction  Decoction  Decoction  Decoction  Crush and press  Decoction  Crush and decoction  Decoction  Decoction  Heat and press  Crush and press  Decoction | Oral  Oral  Oral  Oral  Oral  Oral  Oral  Oral  Oral  Oral  Oral  Oral  Oral  Oral  Oral  Oral  Oral  Oral  Oral  Oral  Oral  Oral  Oral  Oral  Oral  Oral  Oral  Oral  Oral  Oral  Oral  Oral  Oral  Oral  Oral  Oral  Oral  Oral  Oral |
| [146] | Ghana | Public | 53 herbalists and herbal medicines dealers in the Makola, Madina and Nima communities | *Ocimum gratissum* L.  *Moringa oleifera* Lam. | Decoction  Decoction | NR |
| [147] | Ethiopia | Not reported | 100 Sheko informants from the age of 18 and above, drawn from the four kebeles | *Leucas deflexa* Hook.f.  *Rumex nepalensis* Spreng*.* | NR  NR | Oral  Oral |
| [115] | Kenya | Public | Seven women were interviewed to share their knowledge of medicines with us. Their ages ranged between 17 and approximately 65 years (mean 36 years); | *Carissa edulis* (Forssk.) Vahl.  *Aloe vera* (L.) Burm.  *Vernonia amygdalina* Del.  *Capparis fascicularis* DC.  *Gyandropsis gynandra* (L.) Briq*.*  *Maerua calophylla* Gilg.  *Evolvulus alsinoides* (L.) L.  *Ipomoea spathulala* Hall. F.  *Coccinea grandis (L.) Voigt.*  *Euclea racemosa Murr.* ssp. schimperi (A.DC) F.White  *Acacia brevispica Harms*  *Acacia hockii* de Wild  Acacia sp.  *Albizia coriaria* Welw. ex Oliv.  *Ormocarpum trachycarpum* (Taub.) Harms  *Senna bicapsularis* (L.) Roxb.  *Senna dibymobotyra* (Fresen) H.S. Irwin and R.C. Barneby  *Senna occidentalis* (L.) Link  *Leonotis nepetifolia* (L.) Ait.f.  *Ocimum gratissimum* L.  *Sida rhombifolia* L.  *Melia azedarach* L.  *Psidium guajava* L.  *Boerhavia erecta* L.  *Harrisonia abyssinica* Oliv.  *Grewia trichocarpa* Hochst. ex A. Rich.  *Cissus quadrangularis* L.  *Balanites aegyptiaca* (L.) Del. | Decoction  Decoction  Decoction  Decoction  Crush and decoction  Infusion  Decoction  Decoction  Crushed, mixed with water  Decoction  Decoction  Decoction  Crushed, mixed with water  Decoction  Crushed and dried  Crushed  Decoction  Decoction  Decoction  Steambath  Infusion  Infusion  Infusion  Crushed  Infusion and decoction  Decoction  Decoction  Decoction | Oral  Oral  Oral  Oral  Oral  Oral  Oral  Oral  Oral  Oral  Oral  Oral  Oral  Oral  Oral  Oral  Oral  Oral  Oral  Oral  Oral  Oral  Oral  Oral  Oral  Oral  Oral  Oral  Oral |
| [148] | Nigeria | No funding | Interviews with thirty-two (32) clerics. | *Vernonia amygdalina Delile*  *Ocimum gratissimum* L. | Extract juice  Boiling | Oral  Oral |
| [149] | Cameroon | Not reported | A total of 248 respondents were interviewed and 133 medicinal plants belonging to 59 families identified and documented. | *Alstonia boonei* De Willd.  *Gambeya africana (Bak.)* Pierre.  *Hibiscus noldae* Bak. f.  *Phyllanthus muellerianus* (O. Ktze.) Excell.  *Rauwolfia vomitoria* Afzel.  *Rubus fruticosus* Linn.  *Sapium ellipticum Hochst.* ex Krauss Pax  *Trifolium baccarina* Chiov. | Decoction  Decoction  Maceration  Decoction and infusion  Decoction  Infusion  Bark is chewed twice daily  Fried paste is mixed with salt | Oral  Oral  Oral  Oral  Oral  Oral  Oral  Oral |
| [150] | Kenya | Not reported | We interviewed a total of 45 herbalists aged between 38 and 70 years, with majority being female (28). | *Plectranthus barbatus* var. grandis (L.H. Cramer) Lukhoba&A.J. Pat on  *Pseudarthria hookeri* Wight&Arn.  *Syzygium cordatum* Hochst.exC.Kraus | Boiling  Boiling  Boiling | Oral  Oral  Oral |
| [151] | Mauritius | Not reported | A total of 113 local knowledgeable persons and 9 traditional healers (herbalists) from 17 sites from 2014 to 2015 were interviewed | *Ayapana triplinervis* (Vahl) R.M. king et H. Rob.  *Euphorbia hirta* L.  *Desmodium triflorum* (L.) DC.  *Psidium guajava* L.  *Phyllanthus urinaria* L.  *Punica granatum* L. | Chew 1-2 leaves and ingest the juice or prepare an infusion of 1-2 leaves (take 1 cup per day)  Prepare an infusion of 3 leaves in 1L water (take 1-2 cups per day)  Prepare an infusion of 3 whole plants in 1L water, drink 1 throughout the day  Prepare an infusion of 3 buds in 1L of water (take 1-2 cups per day)  Prepare an infusion of 3 whole plants in 1L water (take 1-2 cups per day)  Prepare a decoction of the 1 immature fruit in 1L of water (take 1-2 cups per day) | Oral  Oral  Oral  Oral  Oral  Oral |
| [152] | Ethiopia | Public | 150 Kara (female = 48, male = 112) and 56 Kwego  (female = 20, male = 36). The age of the Kara female informants  ranged from 20 to 70 years (median = 43) | *Hypoestes forskaolii (Vahl)* R. Br.  *Achyranthes aspera L.*  *Aerva javanica* (Burm.f.) Schultes  *Amaranthus spinosus L.*  *Pupalina micrantha* Hauman  *Uvaria leptocladon* Oliv  *Saba comorensis* (Boj.) Pichon  *Balanites rotundifolia* (van Tieghem) Blatter  *Boscia coriacea* Pax  *Cadaba forinosa Forssk.*  *Cissampelos pareira L.*  *Plumbago zeylanica L.*  *Cenchrus ciliaris*  *Lecaniodiscus fraxinifolius* Bak  *Solanum hastifolium Hochst.* ex Dunal  *Solanum incanum L.*  *Withania somnifera (L.)* Dunal  *Cissus rotundifolia* (Forssk.) Vahl | Chewing/infusion  Pounded/infusion  Crushed/mixed with water/infusion  Pounded/mixed water  Chewing/powder mixed with water  Crushed/decoction/boiled with coffee seeds’ husk  Crushed  Crushed  Chewing  Crushed boiled in water  Crushed/powdered boiled with coffee seeds’ husk  Chewing  Crushed/infusion  Chewing  Infusion  Crushed with water  Crushed/infusion | Oral  Oral  Oral  Oral  Oral  Oral  Oral  Oral  Oral  Oral  Oral  Oral  Oral  Oral  Oral  Oral  Oral  Oral |
| [153] | Ethiopia | Not reported | Interviews conducted with 150 informants. In the five  kebeles, 30 informants (15 males and 15 females) were selected | *Aloe spp.*  *Balanites aegyptiaca (L.)* Delile  *Malva parviflora* L.  *Mentha piperita* L. | Cutting to harvest the jelly juice  Crushing to collect juice  Pounding  Pounding after mixing it with *Nigella sativa* and *A. sativum* | Oral  Oral  Oral  Oral |
| [154] | Mauritius | Not reported | interviews were conducted with Mauritians and herbalists (n=307) | *Daucus carota* L.  *Cocos nucifera* L.  *Eupatorium triplinerve* Vahl.  *Bidens pilosa* L.  *Phyllanthus* L.  *Thymus vulgaris* L.  *Maranta arundinacea* L.  *Musa acuminate* Colla  *Psidium guajava* L.  *Hordeum vulgare* L.  *Triticum monococcum* L.  *Punica granatum* L.  *Camellia sinensis* L. | Prepare a decoction followed by addition of sugar and salt (drink 1 cup once daily)  Decoction (drink 1 glass once daily, preferably in the morning)  Decoction (administer 1 tablespoon twice daily)  Decoction (administer 1 glass daily)  Decoction (drink a small cup once daily)  Decoction of *Thymus vulgaris L.* and *Eupatorium triplinerve* Vahl. Leaves (drink 1 glass once daily)  Mix powdered arrow root in water (drink one glass once daily)  Apply warm oil on leaf and bind it on forehead  Decoction (take 1 cup once daily)  Mix 1 teaspoon of powdered barley and 1 pinch of salt and sugar in a glass of water (drink at night before sleeping)  Mix 2 tablespoon of plain flour in a glass of cold water (drink twice daily)  Prepare a decoction of mesocarp of fruit (drink 1 cup once daily)  Decoction of the leaves with addition of 1 tablespoon of *Triticum monococcum* L. (drink one cup in the morning) | Oral  Oral  Oral  Oral  Oral  Oral  Oral  Oral  Oral  Oral  Oral  Oral  Oral |
| [155] | Kenya | Not reported | A total of Sixty (60) traditional health practitioners, aged between 18 and 85 years were interviewed | *Toddalia asiatica* | Concoction | Oral |
| [20] | Mozambique | Public | 15 interviews were carried out in the three markets (ipamanine, Xiquelene and Mazambane) | *Acridocarpus natalitius* A. Juss  *Aloe marlothii* A. Berge  *Combretum molle R.Br.* ex G. Don.  *Elephantorrhiza elephantina* (Burch.) Skeels  *Garcinia livingstonei* T. Anderson  *Gladiolus* sp.  *Gymnosporia heterophylla* (Eckl. & Zeyh.) Loes  *Hugonia orientalis* Engl.  *Hypoxis hemerocallidea Fisch.,* C.A. Mey. & Avé-Lall.  *Kedrostis* sp.  *Phyllanthus*  *Secamone punctulata* Decne.  *Senna occidentalis* (L.) Link  *Spirostachys africana* Sond.  *Strychnos henningsii Gilg*  *Tabernaemontana elegans* Stapf  *Terminalia sericea Burch.* ex DC.  *Tiliacora funifera* (Miers) Oliv. | Decoction  Topical (cover or wash)  Decoction  Decoction  Decoction  Decoction  Decoction  Decoction  Decoction or cold infusion  Decoction or cold infusion  Decoction  Decoction  Decoction  Decoction  Cold infusion or decoction  Decoction  Decoction  Decoction | Oral  Oral  Oral  Oral  Oral  Oral  Oral  Oral  Oral  Oral  Oral  Oral  Oral  Oral  Oral  Oral  Oral  Oral |
| [156] | Uganda | Public | interviewing 205 respondents (117 females and 88 males) from 72 households | *Psidium guajava L.*  *Priva adhaerens* (Forssk.) Chiov | Decoction  Infusion mixed with clay water | Oral  Oral |
| [157] | Mali | Not reported | interviews with 78 healers in three districts in Mali (Siby, Dioïla and Dogonland) | *Terminalia macroptera* | Decoction of root (drink one glass) daily until improvement, decoction of inner root bark (one tea glass, to be drunk once daily for 3 days) | Oral |
| [158] | Tanzania | Not reported | A total of 10 midwives and 40 traditional healers, making a total of 50 respondents, were interviewed. | *Chassalia pavijidia*  *Psidium guajave* | Boiling  Crush and add water | Oral  Oral |
| [159] | Swaziland | Public | Traditional medical practitioners in the region | *Psidium guajave* and *Albizia adianthifolia* (Schumach.) W.F. Wight  *Syzygium guineense* (Willd.) DC. | Infusion (drink 1 tablespoon thrice daily for 3 days)  Concoction (1 tablespoon of to an adult and 1 teaspoon to a child three times daily until diarrhoea stops) | Oral  Oral |
| [120] | Angola | Public | In total, 10 knowledgeable informants (herbalists) were individually interviewed: 3 men/2 women in Kuito and 2 men/3 women in Cuemba. | *Albizia adianthifolia* (Schuma ch.) W.F Wight  *Bridelia sp.*  *Cassytha pondoensis Engl.*  *Diospyros batocana* Hiern.  *Diplorhynchus condylocarpon* (Müll. Arg.) Pichon.  *Eriosema affine* De Wild*.*  *Garcinia huillensis Welw. ex Oliv.*  *Gymnosporia senegalensis* (Lam.) Loes.  *Hymenocardia acida* Tul.  *Ochna pulchra* Hook.  *Oxygonum pachybasis* MilneRedh.  *Parinari capensis* Harv.  *Psorospermum febrifugum* Spach*.*  *Rhynchosia minima (L.) DC.*  *Securidaca longepedunculata* Fresen.  *Syzygium guineense* (Willd.) DC.  *Uapaca gossweileri* Hutch.  *Vangueriopsis lanciflora* (Hiern) Robyns.  *Xylopia tomentosa* Exell*.* | Infusion with tepid water  Decoction  Decoction  Infusion  Infusion  Decoction  Decoction  Decoction  Decoction  Infusion  Decoction  Decoction  Decoction  Decoction  Decoction  Decoction  Decoction and infusion  Infusion with tepid water  Decoction | Oral  Oral  Oral  Oral  Oral  Oral  Oral  Oral  Oral  Oral  Oral  Oral  Oral  Oral  Oral  Oral  Oral  Bath  Oral |
| [35] | South Africa | Public | Interviews were conducted amongst homestead inhabitant | *Acacia burke*  *Acanthospermum glabratum*  *Brachylaena transvaalensis*  *Catharanthus roseus*  *Chenopodium ambrosioides*  *Cissampelos hirta*  *Garcinia livingstonei*  *Gymnosporia senegalensis*  *Krauseola mosambicina*  *Lippia javanica*  *Mangifera indica*  *Melia azedarach*  *Psidium guajava*  *Sarcostemma viminale subsp*. viminal  *Schotia brachypetala*  *Sclerocarya birrea subsp.* Caffra  *Senna occidentalis*  *Strychnos madagascariensis*  *Syzygium cordatum*  *Terminalia sericeaa*  *Trichilia emetic*  *Vangueria infausta subsp.* Infausta  *Vernonia natalensis* | Crushed and infusion (administered twice a day  Infusion, crushed (drunk three times a day by children and 60 ml times a day by adults)  Crushed and infusion (drunk twice a day)  Crushed and infusion. A teaspoon of the infusion is drunk twice a day.  Infusion and crushe (take two times a day)  Infusion (crushed and mixed with cold water). Administered twice a day.  Crushed and Infusion (drunk three times a day)  Infusion (take three times a day)  Crushed, a quarter of a cup is taken two times a day  Crushed and infusion (drunk twice a day).  Infusion (60 ml drunk twice a day)  Crushed and infusion (drunk twice a day)  Crushed and infusion (drunk twice a day)  Crushed and infusion (drunk twice a day)  Crushed and infusion (125 ml drunk three times a day)  Crushed and infusion (drunk twice a day)  Crushed and infusion (drunk twice a day)  Crushed and infusion (60 ml drunk three times a day)  Crushed and infusion. The infusion (60 ml) is drunk twice a day  Crushed and infusion (administered twice a day)  Crushed and infusion (250 ml is administered three times a day  Crushed and infusion ( 60 ml is drunk twice a day) until diarrhoea subsides | Oral  Oral/anal  Oral/anal  Oral  Oral  Oral  Oral/anal  Oral  Oral  Oral  Oral  Oral  Oral  Oral  Oral/anal  Oral  Oral  Oral  Oral/anal  Oral  Oral  Oral |
| [160] | Ethiopia | Public | 50 informants (37 male and 13 female). Among the 50 informants, 15 were key informants (traditional healers) selected with the assistance of clan leaders, peasant association leaders and members of the local community | *Acacia albida* Del.  *Acacia tortilis* (Forssk.) Hayne  *Hydnora johannis Becc.* | Concoction and crushed  Concoction and hot infusion | Oral/anal  Oral  External/oral |
| [161] | South Africa | Not reported | 51 healers from 17 municipalities covering Capricorn, Sekhukhune and Waterberg districts in the Limpopo Province, South Africa were interviewed | *Sclerocarya birrea* (A. Rich.) Hochst.  *Ozoroa sphaerocarpa* R. and A. Fern.  *Carissa edulis (Forssk.)* Vahl*.*  *Dicoma anomala* Sond.  *Helichrysum caespititium* Sond.  *Commiphora marlothii* Engl*.*  *Gymnosporia senegalensis* (Lam.) Exell  *Sansevieria hyacinthoides* (L.) Druce  *Acacia senegal* (L.) Willd*.*  *Vigna unguiculata* (L.) Walp.  *Punica granatum* L.  *Grewia bicolor* Juss.  *Triumffeta* spp.  *Psidium guajava L.*  *Ximenia americana L.*  *Saccharum officinarum L.*  *Osyris lanceolata Hochst. and Steud. ex A. DC.*  *Pappea capensis Eckl. and Zeyh.*  *Solanum lycopersicum L.*  *Dombeya rotundifolia (Hochst.) Planch.* | Hot Infusion (administered thrice a day)  Cooked in water for 5–20 min. The infusion is administered thrice a day  Cooked in water for 10 min. The infusion is administered thrice a day  One tuber is macerated in warm water for 24 h. The infusion is administered thrice a day  Cooked in water for 5–20 min. The infusion is administered thrice a day  Pounded and mixed with warm The infusion is administered thrice a day  Cooked in water for 5–20 min. The infusion is administered thrice a day  Cooked in water for 5 min. The infusion is administered thrice a day  Pounded and mixed with warm water. The infusion is administered thrice a day  Cooked in water for 20 min The infusion is administered thrice a day  Pericarp mixed with *Psidium guajava* roots and cooked in water for 5–25 min The infusion is administered thrice a day  Cooked in water for 5–30 min. The infusion is administered thrice a day  Cooked in water for 5–20 min. The infusion is administered thrice a day  *Punica granatum* roots or pericarp and cooked in water for 5 min. The infusion is administered thrice a day  Cooked in water for 5 min. The infusion is administered thrice a day  Cooked in water for 20 min. The infusion is administered thrice a day  Cooked in water for 5 min or pounded with warm water. The infusion is administered thrice a day  One fruit taken as raw, thrice a day  Juice taken, thrice a day  Cooked in water for 5–20 min. The infusion is administered thrice a day | Oral  Oral  Oral  Oral  Oral  Oral  Oral  Oral  Oral  Oral  Oral  Oral  Oral  Oral  Oral  Oral  Oral  Oral  Oral  Oral |
| [162] | Uganda | Public | 35 respondents were interviewed | *Brachiaria decumbens* Stapf.  *Cajanus cajan* (L.) Millsp.  *Coffea canephora* Pierre ex A. froehner  *Crassocephalum montuosum*  (S. Moore) Milne-Redh.  *Combretum paniculatum* Vent.  *Cordia millenii* Bak.  *Desmodium repandum* (Vahl)  *Desmodium uncinatum* (Jacq.) DC.  *Drypetes battiscombei* Hutch  *Hoslundia opposita* Vahl.  *Ipomoea batatas* (L.) Lam.  *Kosteletzkya adoensis* Mast.  *Lantana trifolia* L.  *Markhamia lutea* K. Schum.  *Passiflora edulis* Sims.  *Paullinia pinnata* L.  *Pseudarthria hookeri* Wight& Arn.  *Rumex usambarensis* (Eng.ex Damm.)  *Triumfetta rhomboidea* Jacq. | Chew or decoction drunk. Squeeze, add water & drink  Pound and add water. 500 ml given once  Infusion with passion fruit, *Coffea canephora* and *Brachiaria decumbens*  Infusion. 500 ml given two times a day for adults for five days  Decoction  Squeeze  Decoction bathed or smear  Infusion  Decoction  Pound with *Passiflora edulis, Coffea canephora* add water  Squeeze & drink  Decoction. 500 ml given three times a day  Pound and add water  Pound and add water  Decoction  Infusion, mixed with *C. cajans* for diarrhoea. 500 ml given three times for one day  Squeeze, add water and drink  Pound and add water | Oral  Oral  Oral  Oral  Oral  Oral  Oral  Oral  Oral  Oral  Oral  Oral  Oral  Oral  Oral  Oral  Oral  Oral  Oral  Oral |
| [163] | South Africa | Not reported | 52 traditional healers residing in the 17 local municipalities within the three districts of the Limpopo Province | *Psidium guajava* L.  *Punica granatum* L. | Mixed with *Punica granatum* (root) and boiled in water for 5 min. Boiled in water for 5–10 min and one tin cup of the extract is taken thrice a day  Boiled in water for 5–25 min and one tin cup of the extract is taken thrice a day | Oral  Oral |
| [164] | Mauritius | Not reported | 52 key Chinese informants among Sino-Mauritians | *Cassytha filiformis*  *Myristaca fragrans*  *Verbena officinalis*  *Alpinia oxyphylla*  *Zingiber officinalis* | Decoction  Decoction  Infusion  Infusion  Infusion | Oral  Oral  Oral  Oral  Oral |
| [117] | Ethiopia | Not reported | A total of 38 informants constituted of professional healers and knowledgeable farmers with the ages of 25 and above were interviewed. Of the total, 16 were Agew-Awis, 11 were Shinashas and 11 were Amharas. | *Calpurnia aurea* (Ait.) Benth.  *Cordia africana* Lam.  *Stereospermum kunthianum* Cham.  *Verbena officinalis L.* | NR  NR  NR  NR | Oral  Oral  Oral  Oral |

Minimum inhibitory concentration: MIC, Minimum Bactericidal Concentration: MBC, Not reported: NR

**Studies included** (n = 27)

***In vivo* studies** (n = 7)

***In vitro* Studies** (n = 11)

***In vitro & in vivo* studies** (n = 2)

**Plant species with good activity**

(n = 12)

*Dodonaea viscosa, Psidium guajava, Acacia nilotica, Parkia biglobosa, Anacardium occidentale, Vernonia amygdalina, Pterocarpus erinaceus, Senna italic, Manihot esculenta,* *Khaya senegalensis,* *Ocimum gratissimum,* and *Diospyros mespiliformis*

***In vitro* Studies & Ethnobotanical** (n = 4)

**Plant species with good activity** (n = 18)

*Whitfieldia elongate, Bidens pilosa, Hypericum roeperianum, Acacia burke, Sclerocarya birrea, Canarium schweinfurthii, Brachylaena transvaalensis, Terminalia sericea, Strombosia scheffleri, Garcinia livingstonei, Dissotis brazzae, Erythrina caffra, Psidium guajava, Mangifera indica*

**Combinations:**

*Acanthospermum glabratum + Krauseola mosambicina,*

*Psidium guajava + A. glabratum, Brachylaena transvaalensis + P. guajava, Sclerocarya birrea + B. transvaalensis*

**Plant species** **with positive results** (n = 2)

*Nymphaea lotus* and *Cola anomala*

**Plant species with good activity, *in vitro***

(n = 10)

*Searsia chirindensis, Punica granatum, Indigofera daleoides, Maesa lanceolate, Psidium guajava, Hypoxis hemerocallidea, Eucalyptus camaldulensis, Solanum aculeastrum, Rapanea melanophloeos,* and *Ficus craterostoma*

**Plant species with moderate activity** (n = 1)

*Ozoroa insignis*

**Plant species with moderate activity** (n = 6)

*Vitex doniana, Osyris quadripartite, Daniellia oliveri, Citrus limon, Priva adhaerens,* and *Rauvolfia vomitoria*

**Plant species with poor (least) activity,**

***in vitro*** (n = 10)

*Elephantorrhiza elephantina, Elephantorrhiza burkei, Ximenia caffra, Schotia brachypetala*

*Syzygium cordatum, Gymnosporia senegalensis, Ozoroa insignis, Ximenia caffra,*

*Spirostachys Africana, Hydnora africana,*

*Clutia pulchella, Dais cotinifolia, Tecoma capensis,* and *Trema orientalis*

**Plant species with moderate activity** (n = 7)

*Maytenus peduncularis, Artocarpus heterophyllus Cremaspora triflora, Maytenus procumbens, Pelargonium sidoides DC., Maytenus senegalensis, Dichrostachys cinerea*

**Plant species with poor (least) activit** (n = 1)

*Acanthospermun hispidum*

**Plant species with poor (least) activity** (n = 23)

*Vernonia natalensis, Acacia mearnsii, Aloe striata, Catharanthus roseus, Hydnora Africana Chenopodium ambrosioides, Cissampelos hirta, Gymnosporia senegalensis, Hermbstaedtia odorata, Hypoxis latifolia, Isoglosa lacteal, Krauseola mosambicina, Lippia javanica, Maytenus undata, Melia azedarach, Sarcostemma viminale, Schizocarphus nervosus, Schotia brachypetala, Senna occidentalis, Strychnos madagascariensis, Syzygium cordatum, Trichilia emetic, Vangueria infausta*

**Plant species with toxic effects** (n = 4)

*Elaeodendron croceum***,** *Hydnora johannis (low), Calpurnia aurea***,** *Maesa lanceolata*

**Supplementary Figure S1:** Summary of included studies reporting the plant species with good, moderate, and/or least activity for diarrhoea treatment.

: Plant species with positive and negative effects in treatment of diarrhoea.

**References**

12. Walusansa A, Asiimwe S, Ssenku J, Anywar G, Namara M, Nakavuma JL, Kakudidi EK. Herbal medicine used for the treatment of diarrhea and cough in Kampala city, Uganda. Trop Med Int Health. 2022;50(1):1-21. <https://doi.org/10.1186/s41182-021-00389-x>.

20. Lulekal E, Asfaw Z, Kelbessa E, Van Damme P. Ethnomedicinal Study of Plants Used for Human Ailments in Ankober District, North Shewa Zone, Amhara Region, Ethiopia. J Ethnobiol Ethnomed. 2013;9:63. <https://doi.org/10.1186/1746-4269-9-63>.

35. de Wet H, Nkwanyana MN, van Vuuren SF. Medicinal plants used for the treatment of diarrhoea in northern Maputaland, KwaZulu-Natal Province, South Africa. J Ethnopharmacol. 2010;130(2):284-289. https://doi.org/10.1016/j.jep.2010.05.004.

54. Wambe H, Noubissi PA, Fokam Tagne MA, Foyet Fondjo A, Fankem GO, Kamtchouing I, Ngakou Mukam J, Nguelefack TB, Kamgang R. Anti-shigellosis activity of *Cola anomala* water/ethanol pods extract on *Shigella* *flexneri*-induced diarrhea in rats. BioMed Res Int. 2019. <https://doi.org/10.1155/2019/6706230>.

56. Yagi S, Chrétien F, Duval RE, Fontanay S, Maldini M, Piacente S, Henry M, Chapleur Y, Laurain-Mattar D. Antibacterial activity, cytotoxicity and chemical constituents of *Hydnora johannis* roots. S Afr J Bot. 2011;78:228-234. <https://doi.org/10.1016/j.sajb.2011.09.010>.

58. Otshudi AL, Vercruysse A, Foriers A. Contribution to the ethnobotanical, phytochemical and pharmacological studies of traditionally used medicinal plants in the treatment of dysentery and diarrhoea in Lomela area, Democratic Republic of Congo (DRC). J Ethnopharmacol. 2000;71(3):411-423. https://doi.org/10.1016/s0378-8741(00)00167-7.

59. Maroyi A. An ethnobotanical survey of medicinal plants used by the people in Nhema communal area, Zimbabwe. J Ethnopharmacol. 2011;136(2):347-354.

61. Omwenga E, Okemo PO, Mbugua PK, Ogol C. Ethnobotanical survey and antimicrobial evaluation of medicinal plants used by the Samburu community (Kenya) for treatment of diarrhoea. Phcog Mag. 2009;5(18):165-175.

65. Bisi-Johnson MA, Obi CL, Samuel BB, Eloff JN, Okoh AI. Antibacterial activity of crude extracts of some South African medicinal plants against multidrug resistant etiological agents of diarrhea. BMC Complement Altern Med. 2017;17(1):1-9.

72. Elisha IL, Botha FS, McGaw LJ, Eloff JN. The antibacterial activity of extracts of nine plant species with good activity against *Escherichia coli* against five other bacteria and cytotoxicity of extracts. BMC Complement Altern Med. 2017;17(1):1-10.

73. Abdela J. Evaluation of *in vivo* antidiarrheal activities of hydroalcoholic leaf extract of *Dodonaea viscosa* L. (*Sapindaceae*) in Swiss albino mice. Evid Based Complement Alternat Med. 2019;24.2515690X19891952. <https://doi.org/10.1177/2515690X19891952>.

74. Dougnon TV, Hounsa E, Agbodjento E, Koudokpon H, Legba B, Fabiyi K, Afaton A, Sintondji K, Akpode B, Klotoé JR, Tchobo F. Toxicological Characterization of Ten Medicinal Plants of the Beninese Flora Used in the Traditional Treatment of Diarrheal Diseases. J Evid Based Complementary Altern Med. 2021. <https://doi.org/10.1155/2021/6676904>.

75. Nansunga M, Barasa A, Abimana J, Alele PE, Kasolo J. Safety and antidiarrheal activity of *Priva adhaerens* aqueous leaf extract in a murine model. J Ethnopharmacol. 2014;157:251-256. https://doi.org/10.1016/j.jep.2014.09.044.

76. Bello FH, Maiha BB, Anuka JA. The effect of methanol rhizome extract of *Nymphaea lotus* Linn. (Nymphaeaceae) in animal models of diarrhea. J Ethnopharmacol. 2016;190:13-21. <https://doi.org/10.1016/j.jep.2016.05.036>.

87. van Vuuren SF, Nkwanyana MN, de Wet H. Antimicrobial evaluation of plants used for the treatment of diarrhoea in a rural community in northern Maputaland, KwaZulu-Natal, South Africa. BMC Complement Altern Med. 2015;15(1):1-8. https://doi.org/10.1186/s12906-015-0570-2.

89. Kareru PG, Kenji GM, Gachanja AN, Keriko JM, Mungai G. Traditional medicines among the Embu and Mbeere people of Kenya. Afr J Tradit Complement Altern Med. 2007;4(1):75-86. https://doi.org/10.4314/ajtcam.v4i1.31193.

96. Ngonda F, Magombo Z, Mpeketula P, Mwatseteza J. Extraction, characterization, and pharmacological evaluation of leaves and root bark of *Dalbergiella nyasae* (Baker f.). Pharmacogn J. 2012;4(34):69-76.

97. Moshi MJ, Innocent E, Masimba PJ, Otieno DF, Weisheit A, Mbabazi P, Lynes M, Meachem K, Hamilton A, Urassa I. Antimicrobial and brine shrimp toxicity of some plants used in traditional medicine in Bukoba District, north-western Tanzania. Tanzan J Health Res. 2009;11(1).

104. Gumisiriza H, Sesaazi CD, Olet EA, Kembabazi O, Birungi G. Medicinal plants used to treat" African" diseases by the local communities of Bwambara sub-county in Rukungiri District, Western Uganda. J Ethnopharmacol. 2021;268:113578. https://doi.org/10.1016/j.jep.2020.113578.

109. Mwambete KD, Joseph R. Knowledge and perception of mothers and caregivers on childhood diarrhea and its management in Temeke municipality, Tanzania. Tanzan J Health Res. 2010;12(1):47-54. <https://doi.org/10.4314/thrb.v12i1.56278>.

115. Geissler PW, Harris SA, Prince RJ, Olsen A, Achieng'Odhiambo R, Oketch-Rabah H, Madiega PA, Andersen A, Mølgaard P. Medicinal plants used by Luo mothers and children in Bondo district, Kenya. J Ethnopharmacol. 2002;83(1-2):39-54. https://doi.org/10.1016/s0378-8741(02)00191-5.

116. Giday K, Asfaw Z, Woldu Z. Medicinal plants of the Meinit ethnic group of Ethiopia: An ethnobotanical study. J Ethnopharmacol. 2009;85(1):1-11.

117. Giday M, Teklehaymanot T, Animut A, Mekonnen Y. Medicinal plants of the Shinasha, Agew-awi and Amhara peoples in northwest Ethiopia. J Ethnopharmacol. 2007;110(3):516-525. https://doi.org/10.1016/j.jep.2006.10.011.

118. Ribeiro A, Romeiras MM, Tavares J, Faria MT. Ethnobotanical survey in Canhane village, district of Massingir, Mozambique: medicinal plants and traditional knowledge. J Ethnobiol Ethnomedicine. 2010;6(1):1-15. <https://doi.org/10.1186/1746-4269-6-33>.

120. Novotna B, Polesny Z, Pinto-Basto MF, Van Damme P, Pudil P, Mazancova J, Duarte MC. Medicinal plants used by ‘root doctors,’ local traditional healers in Bié province, Angola. J Ethnopharmacol. 2020;260:112662. https://doi.org/10.1016/j.jep.2020.112662.

121. Aparicio H, Hedberg I, Bandeira S, Ghorbani A. Ethnobotanical study of medicinal and edible plants used in Nhamacoa area, Manica province–Mozambique. S Afr J Bot. 2021;139:318-328. https://doi.org/10.1016/j.sajb.2021.02.029.

125. Eve A, Aliero AA, Nalubiri D, Adeyemo RO, Akinola SA, Pius T, Nabaasa S, Nabukeera S, Alkali B, Ntulume I. *In vitro* antibacterial activity of crude extracts of *Artocarpus heterophyllus* seeds against selected diarrhea-causing superbug bacteria. Sci World J. 2020. <https://doi.org/10.1155/2020/9813970>.

126. Ahmed AS, McGaw LJ, Eloff JN. Evaluation of pharmacological activities, cytotoxicity and phenolic composition of four Maytenus species used in southern African traditional medicine to treat intestinal infections and diarrhoeal diseases. BMC Complement Altern Med. 2013;13(1):1-15. <https://doi.org/10.1186/1472-6882-13-100>.

127. Ngoci SN, Matasyoh JC, Mwaniki CG, Mwendia CM. Antibacterial activity of methanol root extract of *Indigofera lupatana* Baker F. East J Med. 2012;17(1):11-16.

128. Teferi MY, Abdulwuhab M, Yesuf JS. Evaluation of *in vivo* antidiarrheal activity of 80% methanolic leaf extract of *Osyris quadripartita Decne* (*Santalaceae*) in Swiss Albino Mice. Evid Based Complement Alternat Med. 2019;24:2515690X19833340. https://doi.org/10.1177/2515690X19833340.

129. Guadie A, Dakone D, Unbushe D, Wang A, Xia S. Antibacterial activity of selected medicinal plants used by traditional healers in Genta Meyche (Southern Ethiopia) for the treatment of gastrointestinal disorders. Braz J Med Biol Res. 2020;22:100338.

130. Madikizela B, Ndhlala AR, Finnie JF, Van Staden J. Ethnobotanical, phytochemical and pharmacological analysis of medicinal plants used for the management of diarrhoea in Bizana, Eastern Cape Province, South Africa. J Ethnopharmacol. 2012;141(1):61-74.

131. Olajuyigbe OO, Afolayan AJ. *In vitro* antibacterial and time-kill evaluation of *the Erythrina Caffra* Thunb. Extract against bacteria associated with diarrhoea. Pharm Biol. 2012;50(2):230-235.

132. Shandukani PD, Tshidino SC, Masoko P, Moganedi KM. Antibacterial activity and in situ efficacy of *Bidens pilosa* Linn and *Dichrostachys cinerea* Wight et Arn extracts against common diarrhoea-causing waterborne bacteria. Evid Based Complement Alternat Med. 2018;2018:9424309.

133. Ojewole JA, Amabeoku GJ, Kabatende J. Antidiarrhoeal activity of *Psidium guajava* Linn. (*Myrtaceae*) leaf aqueous extract in rodents. J Smooth Muscle Res. 2008;44(6):195-207.

134. Agunu A, Abdurahman EM, Andrew GO, Muhammed Z, Chindo BA, Gamaniel KS. Effect of acute and sub-chronic administration of methanol leaf extract of *Gmelina arborea* on haematological and serum lipid profile of rats. J Ethnopharmacol. 2005;101(1-3):275-284.

135. Adeniyi OS, Omale J, Omeje SC, Edino VO. Antidiarrheal activity of hexane extract of *Citrus limon* peel in an experimental animal model. J Integr Med. 2017;15(2):158-64.

136. Ajibesin KK, Ekpo BA, Bala DN, Essien EE, Adesanya SA. Ethnobotanical survey of Akwa Ibom State of Nigeria. J Ethnopharmacol. 2008;115(3):387-408.

137. Asowata-Ayodele AM, Afolayan AJ, Otunola GA. Ethnobotanical survey of culinary herbs and spices used in the traditional medicinal system of Nkonkobe Municipality, Eastern Cape, South Africa. S Afr J Bot. 2016;104:69-75.

138. Mugomeri E, Chatanga P, Raditladi T, Makara M, Tarirai C. Ethnobotanical study and conservation status of local medicinal plants: Towards a repository and monograph of herbal medicines in Lesotho. Afr J Tradit Complement Altern Med. 2016;13(1):143-156.

139. Tshikalange TE, Mophuting BC, Mahore J, Winterboer S, Lall N. An ethnobotanical study of medicinal plants used in villages under Jongilanga tribal council, Mpumalanga, South Africa. Afr J Tradit Complement Altern Med. 2016;13(6):83-89.

140. Van Wyk BE, De Wet H, Van Heerden FR. An ethnobotanical survey of medicinal plants in the southeastern Karoo, South Africa. S Afr J Bot. 2008;74(4):696-704.

141. Suleiman MH. An ethnobotanical survey of medicinal plants used by communities of Northern Kordofan region, Sudan. J Ethnopharmacol. 2015;176:232-242.

142. Wondimu T, Asfaw Z, Kelbessa E. Ethnobotanical study of medicinal plants around 'Dheeraa' town, Arsi Zone, Ethiopia. J Ethnopharmacol. 2007;112(1):152-161.

143. Mahwasane ST, Middleton L, Boaduo N. An ethnobotanical survey of indigenous knowledge on medicinal plants used by the traditional healers of the Lwamondo area, Limpopo province, South Africa. South African Journal of Botany. 2013;88:69-75.

144. Chifundera K. Contribution to the inventory of medicinal plants from the Bushi area, South Kivu Province, Democratic Republic of Congo. Fitoterapia. 2001;72(4):351-368.

145. Rakotoarivelo NH, Rakotoarivony F, Ramarosandratana AV, Jeannoda VH, Kuhlman AR, Randrianasolo A, Bussmann RW. Medicinal plants used to treat the most frequent diseases encountered in Ambalabe rural community, Eastern Madagascar. J Ethnobiol Ethnomed. 2015;11:1-6.

146. Bekoe EO, Agyare C, Boakye YD, Baiden BM, Asase A, Sarkodie J, Nettey H, Adu F, Otu PB, Agyarkwa B, Amoateng P. Ethnomedicinal survey and mutagenic studies of plants used in Accra metropolis, Ghana. J Ethnopharmacol. 2020;248:112309.

147. Giday M, Asfaw Z, Woldu Z. Ethnomedicinal study of plants used by Sheko ethnic group of Ethiopia. J Ethnopharmacol. 2010;132(1):75-85.

148. Alade GO, Kola A. Herbal medicine: Clerics’ knowledge in a sub urban center in Niger Delta, Nigeria-a pilot study. J Pharm Pharmacogn Res. 2017;5(4):200-216.

149. Focho DA, Ndam WT, Fonge BA. Medicinal plants of Aguambu-Bamumbu in the Lebialem highlands, southwest province of Cameroon. Afr J Pharm Pharmacol. 2009 ;3(1):1-3.

150. Kigen G, Maritim A, Some F, Kibosia J, Rono H, Chepkwony S, Kipkore W, Wanjoh B. Ethnopharmacological survey of the medicinal plants used in Tindiret, Nandi County, Kenya. Afr J Tradit Complement Altern Med. 2016;13(3):156-168.

151. Samoisy AK, Mahomoodally F. Ethnopharmacological appraisal of culturally important medicinal plants and polyherbal formulas used against communicable diseases in Rodrigues Island. J Ethnopharmacol. 2016;194:803-818.

152. Teklehaymanot T, Giday M. Quantitative ethnobotany of medicinal plants used by Kara and Kwego semi-pastoralist people in lower Omo River Valley, Debub Omo zone, southern nations, nationalities and peoples regional state, Ethiopia. J Ethnopharmacol. 2010;130(1):76-84.

153. Osman A, Sbhatu DB, Giday M. Medicinal plants used to manage human and livestock ailments in Raya Kobo District of Amhara Regional State, Ethiopia. Evid Based Complement Alternat Med. 2020;2020.

154. Nunkoo DH, Mahomoodally MF. Ethnopharmacological survey of native remedies commonly used against infectious diseases in the tropical island of Mauritius. J Ethnopharmacol. 2012;143(2):548-564.

155. Orwa JA, Jondiko IJ, Minja RJ, Bekunda M. The use of *Toddalia asiatica* (L) Lam.(Rutaceae) in traditional medicine practice in East Africa. J Ethnopharmacol. 2008;115(2):257-262.

156. Ssegawa P, Kasenene JM. Medicinal plant diversity and uses in the Sango bay area, Southern Uganda. J Ethnopharmacol. 2007;113(3):521-540.

157. Pham AT, Dvergsnes C, Togola A, Wangensteen H, Diallo D, Paulsen BS, Malterud KE. *Terminalia macroptera*, its current medicinal use and future perspectives. J Ethnopharmacol. 2011;137(3):1486-1491.

158. Mahonge CP, Nsenga JV, Mtengeti EJ, Mattee AZ. Utilization of medicinal plants by Waluguru people in east Uluguru Mountains Tanzania. 2006.

159. Amusan OO, Dlamini PS, Msonthi JD, Makhubu LP. Some herbal remedies from Manzini region of Swaziland. J Ethnopharmacol. 2002;79(1):109-112.

160. Belayneh A, Asfaw Z, Demissew S, Bussa NF. Medicinal plants potential and use by pastoral and agro-pastoral communities in Erer Valley of Babile Wereda, Eastern Ethiopia. J Ethnobiol Ethnomed. 2012;8(1):1.

161. Semenya SS, Maroyi A. Medicinal plants used by the Bapedi traditional healers to treat diarrhea in the Limpopo Province, South Africa. J Ethnopharmacol. 2012;144(2):395-401.

162. Namukobe J, Kasenene JM, Kiremire BT, Byamukama R, Kamatenesi-Mugisha M, Krief S, Dumontet V, Kabasa JD. Traditional plants used for medicinal purposes by local communities around the Northern sector of Kibale National Park, Uganda. J Ethnopharmacol. 2011;136(1):236-245.

163. Semenya S, Potgieter M, Tshisikhawe M, Shava S, Maroyi A. Medicinal utilization of exotic plants by Bapedi traditional healers to treat human ailments in Limpopo province, South Africa. J Ethnopharmacol. 2012;144(3):646-655.

164. Mahomoodally MF, Muthoorah LD. An ethnopharmacological survey of natural remedies used by the Chinese community in Mauritius. Asian Pac J Trop Biomed. 2014;4:S387-S399.

165. Giday M, Asfaw Z, Elmqvist T, Woldu Z. An ethnobotanical study of medicinal plants used by the Zay people in Ethiopia. J. Ethnopharmacol. 2003;85(1):43-52. https://doi.org/10.1016/s0378-8741(02)00359-8.
